# Process of adaptation, development and assessment of acceptability of a health educational intervention to improve referral uptake by people with diabetes in Sri Lanka

**DOI:** 10.1186/s12889-019-6880-4

**Published:** 2019-05-21

**Authors:** M. M. P. N. Piyasena, Maria Zuurmond, Jennifer L. Y. Yip, G. V. S. Murthy

**Affiliations:** 1 Vitreo-retina unit, National Eye Hospital, Colombo 10, Sri Lanka; 2 Clinical Research Department, International Centre for Eye Health, London School of Hygiene and Tropical Medicine, Keppel Street, London, WC1E 7HT UK; 3 International Centre for Evidence in Disability, Clinical Research Department, London School of Hygiene and Tropical Medicine, Keppel Street, London, WC1E 7HT UK

**Keywords:** Acceptability, Diabetic retinopathy, Health education, Referral, Screening, Sri Lanka

## Abstract

**Background:**

One major barrier to uptake of diabetic retinopathy (DR) services is lack of knowledge and awareness of DR among the people with diabetes (PwDM). Targeted health education (HE) can be a key element in improving the uptake of eye care services. Such interventions are lacking in Sri Lanka.

**Methods:**

A local context specific HE intervention (HEI) was developed by adopting available resources and incorporating views from PwDM and key stakeholders. Four sessions of participatory workshops with PwDM (20 Sinhala and 13 Tamil speaking) and two stage 12 stakeholder interviews were conducted to both develop and pre-test the material. The products were a video and a leaflet, delivered at a medical clinic to a sample of 45 PwDM identified as having DR. Semi-structured interviews were conducted after 4 weeks, to evaluate the acceptability and comprehension of the HEI. Additionally, nine interviews were conducted with clinical providers to explore process issues related to delivery of the HEI. Data analysis was conducted using thematic analysis.

**Results:**

The lack of knowledge and awareness on DR, and of the importance of regular DR screening and follow up, combined with poor information on referral pathways were key elements identified from the workshops with PwDM. The stakeholders prioritised the importance of using simple language, and the need for emphasis on improving understanding about the asymptomatic phase of DR. The overall acceptability of the HEI material was satisfactory, although there was some difficulty with interpretation of medical images. Overall, although PwDM liked the ideas of the video, the leaflet was seen as a more practical option, given the busy clinic environment. The key issue was both formats required interaction with the provider, in order to support understanding of the messages.

**Conclusions:**

The process of adapting HE material is not simply translation into the appropriate language. Instead, a tailored approach in a country, context and particular health services setting is needed. This study illustrates the value of using a participatory approach and involving PwDM and stakeholders in the adaptation and pilot testing of a HEI to improve uptake of screening for DR in the context of Sri Lanka.

**Electronic supplementary material:**

The online version of this article (10.1186/s12889-019-6880-4) contains supplementary material, which is available to authorized users.

## Background

Diabetes mellitus (DM) is a global epidemic. Evidence suggests that there will be an increase in the number of people with DM (PwDM) globally with a higher increase in low and middle income countries (LMIC) [1]. One major complication of DM is diabetic retinopathy (DR) and this leads to visual impairment and blindness if not detected and treated on time. The “St Vincent Declaration” stated that all nations should plan efforts to control the complications of DM in their populations [2]. Despite current efforts, DR screening (DRS) coverage is low in many settings. As an example, in USA only 33–68% of PwDM underwent an annual fundus examination [3] and in UK approximately 80% attended for DRS, following an invitation [4]. Therefore, screening uptake is not even optimal even in high income countries (HICs), despite availability of free services. Inequity had been observed in DRS services delivery, even in HICs [5].

A review conducted on interventions to promote DRS, highlighted the need for strategies to improve PwDM’s awareness on DR [6]. Studies show that low functional health literacy [7], language barriers such as the lack of provision of health education (HE) in local languages and dialects and suitability of the content of provider-patient communication should be considered in effective management of chronic diseases like DM [8, 9]. Uptake of DRS depends on the knowledge, attitude and practice of PwDM [10, 11]. Most studies have shown that lack of knowledge and awareness about DR and poor understanding of the need for regular follow-up are major barriers to access [12, 13]. Further, the asymptomatic early stage of DR is a hindrance to access [14]. Therefore, HE about DR must be a key element of any screening strategy for DR [15].

In Sri Lanka, despite free eye care being provided through the public sector, the uptake of DR services is low. The current method of detecting DR is opportunistic screening by ophthalmologists following referral from general physicians or when PwDM present for other eye problems. The highest recorded prevalence of DM of 18.6% (95% CI 15.8–21.5%) in Sri Lanka is in the Western province [16], where a situational analysis showed a significant level of unmet need [17]. A qualitative research study on barriers to accessing services in the Western region also found that knowledge and awareness of DR and DRS among the PwDM was low (under review; service user perspectives - BMC Tropical Medicine and Health and service provider perspectives - BMC Health Services Research).

Health education forms an essential component of the health promotional activities for chronic diseases such as DM [18]. A review on eye health promotion in low income settings stated that behaviour change HE, improvement of access to services and effective advocacy are the major components to address control of blindness [19]. In this approach, reviewers described HE as the “*back bone*” of the health promotion efforts [19]. One major challenge is the translation of behaviour change principles into practical environments attaining the expected outcome [20]. There are many influences at community level such as culture, economy, traditions and social norms that would affect the behaviour of a person [19]. A systematic review of studies which have assessed effectiveness of interventions to improve DRS stated that increasing PwDM awareness on DR can improve the uptake of DRS [21]. Therefore, provision of information through HE material would be an important component in any health promotional strategy to improve uptake of DRS services.

### Frameworks and guidelines

In the development of any HE intervention (HEI), there are a variety of behaviour change models which can be applied. Social Cognitive Theory (SCT) underpins many approaches to behaviour change [22], and was the underpinning framework for this study. It describes how knowledge of risks and benefits of a health condition is a necessary pre-condition needed for a change. This knowledge is related to observing others, social interactions and experiences. A person’s behaviour is understood to both influence and in turn, is influenced by a range of personal and environmental factors.

The overall purpose of this study was to develop a relevant, acceptable and comprehensible HEI for the Sri Lanka context to improve referral uptake at the ophthalmologist’s clinic. This was part of a larger feasibility study to develop an integrated DRS program in the Western province of Sri Lanka.

## Methods

**Fig. 1:**
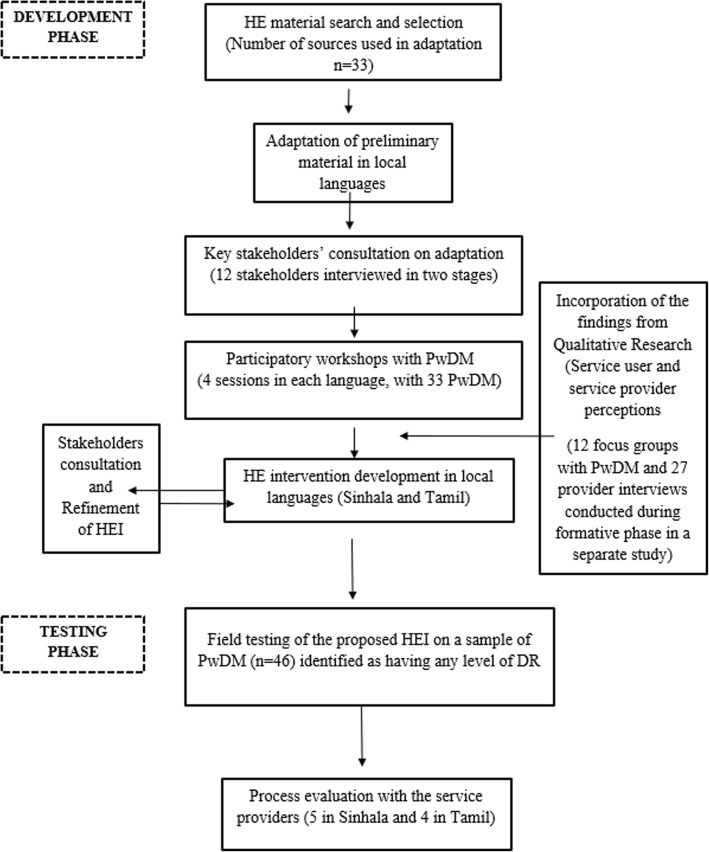
Flowchart of steps in development of HE intervention

It is a descriptive qualitative study which details the process of the development and field testing of a relevant and acceptable HEI. The different phases are described in Fig. 1. For the stages of consultation and revision of the HEI, we adopted a participatory approach, to promote greater acceptance and relevance of the material, in line with general guidance on community based participatory research [23].

### Data collection and analysis

#### Ethics

Ethics approval was obtained from the Ethics Review Committees of the London School of Hygiene and Tropical Medicine-United Kingdom and from the National Eye Hospital-Colombo-Sri Lanka. Written informed consent was obtained from all participants for participation, audio recording and usage of anonymous quotes in publications.

#### Development phase

Initially a search was conducted for existing HE resources over the last 10 years that promoted uptake of DRS or DR services. Details of the electronic data search are provided in Additional file 1. The resources were assessed for comprehensibility and actionability using ‘Patient Education Material Assessment Tool’ (PEMAT) guidelines [24]. A selection of relevant materials was then translated into the two main local languages (Sinhala and Tamil) for reviewing with stakeholders and PwDM.

The second step was one of the two-stage consultation process with 12 key stakeholders who worked in clinical management and health promotion of DM and DR in the public sector (see Table 1). The stakeholders were selected considering their experience and engagement in clinical management of PwDM and involvement of DR related eye care programs. Initially, materials were reviewed to agree on content, culture relevance and suitable mode of delivery for the local context.

**Table 1:** Key stakeholders

Public health sector	
1) Health Education and Promotion Unit – Ministry of Health – Sri Lanka	
2) College of Community Physicians of Sri Lanka	
3) Diabetes Education Unit – National Hospital of Sri Lanka	
4) Vision 2020 Program (DR blindness prevention program) – Ministry of Health – Sri Lanka	
5) Department of Sociology (Medical anthropology)	
6) Media personnel (a newspaper reporter)	
7) A person with diabetes and a person with DR from the Western province (patient representatives)	
Service delivery personnel	
8) Association of Vitreo Retina Specialists of Sri Lanka	
9) College of Ophthalmologists of Sri Lanka	
10) Sri Lanka Optometric Association - Sri Lanka	
11) Ceylon College of Physicians - Sri Lanka	
12) College of Endocrinologists - Sri Lanka	

The next step was running a total of eight participatory workshops with a purposive sample of PwDM in Sinhala (*n* = 10, 4 sessions) and Tamil (n = 10, 4 sessions). The diagnosed PwDM identified at the medical clinic were selected considering gender, main language and to represent various levels of education and income levels. The overall aim was to explore the participants’ views on the selected materials in terms of (1) cultural acceptability of key messages, (2) comprehension, (3) content, (4) layout and design and (5) medium of delivery of the HEI. Details of the participatory workshops are available in Table 2. Final refinement of the material, in terms of the clinical, educational, technical and affective properties of the HEI [25] was then approved at a second meeting with stakeholders. A secondary aim of the meetings was to foster ownership of the developing process of the HEI by key professionals, given they would then be responsible for the implementation of the HEI.

**Table 2:** Schedule of the participatory workshop

Day	Participants	Activity
Day 1	All	Introduced to the research question by main investigator
	Sub groups 1 – Sinhala Subgroup 2 – Tamil	Group work on identifying needs, problems and solutions on accessing services at ophthalmologist’s / retinologist’s clinic following referral (those who identified with referable level DR) from medical clinic – facilitated by moderators
	Sub groups 1 and 2	Exposure to adapted and developed provisional HE interventions – facilitated by moderators
Day 2	Sub groups 1 and 2	Development / modification of HE interventions appropriate to the local context by incorporating participants’ ideas - facilitated by moderators
Day 3	Sub groups 1	Presentation and discussion of findings of assessment of developed HE interventions by participants – facilitated by main investigator with co-moderators.
Day 4	Sub groups 2	

Finally, qualitative research findings, conducted as part of the wider study and published separately (under review), were incorporated into the adaption and development of the HEI. The end result was the production of a video and leaflet, available in two local languages (see Additional file 2 for the leaflets and the videos multi-media files).

#### Data analysis

The participatory workshops and semi-structured interviews (SSIs) were audio recorded and for stakeholders’ meetings detailed notes were taken. A simple thematic analysis was undertaken which included covering themes of comprehension, readability and cultural acceptability. The analysis was conducted in local languages by experienced sociologists. Recordings were transcribed, and together with field notes, were coded in Sinhala and Tamil by local sociologists and then cross checked by the lead sociologist and by the lead investigator. Final themes were translated into English.

#### Field testing phase

The video and leaflet were then field tested at a tertiary level medical clinic setting by administering to a purposive sample of 45 PwDM who had diagnosed any level of DR (see Table 3 for participants’ characteristics). We recruited PwDM in order to equally distribute by gender, main language and ethnic group and those who have already treated for DR were not included. The material was delivered by physician graders in Sinhala, and a trained sociologist in Tamil during the consultation when they presented for out-patient care at the medical clinic. On average video run time was 5 min. It required about 3–5 min to read all pages of the leaflet. First, we shared the leaflet while PwDM were waiting and then the video during the clinical consultation. SSI were then conducted with the PwDM up to 4 weeks later. A purposive sample of service providers (*n* = 9, 5 in Sinhala and 4 in Tamil language) were also interviewed from medical and eye clinics to explore their perspectives on the process of delivery of the HEI. The field-testing interview topic guides details described in Additional file 3.

**Table 3:** Participants characteristics of those who underwent delivering and assessment of HEI

Variable	Results
Mean age (SD)	62.3 years (±9.7)
Mean age at diagnosis of diabetes mellitus (SD)	50.8 years (±8.9)
Mean duration of diabetes mellitus (SD)	11.5 years (±9.0)
Gender	Female 57.8% (26/45)
	Male 42.2% (19/45)
Ethnic group	Sinhalese 53.35% (24/45)
	Tamil 24.4% (11/45)
	Moor 22.2% (10/45)
Main language	Sinhala 53.3% (24/45)
	Tamil 46.7 (21/45)
Residing district	Colombo 93.3% (42/45)
	Gampaha 4.4% (2/45)
	Kalutara 2.2% (1/45)
Level of education	No Schooling 15.6% (7/45)
	Primary (Grade 1 to 5) 31. 1% (14/45)
	Secondary (Grade 6 to 10) 17.85 (8/45)
	Up to GCE O/L (Grade 11) 15.6% (7/45)
	Up to GCE A/L (Grade 12) 17.8% (8/45)
	Degree and above 2.2% (1/45)
Level of monthly income	Low (< £150) 80.0% (36/45)
	Middle (<£300 > £ 150) 8.9% (4/45)
	High (> £300) 11.1% (5/45)
Wearing spectacles at presentation (near or distant)	Had spectacles at presentation 46.7% (21/45)
	Did not have 53.3% (24/45)
Level of diabetic retinopathy	Right eye – No DR 8.9%, any DR 91.1%
	Left eye – No DR 11.1%, any DR 88.9%

## Results

In the developmental phase, a total of 96 HE resources were initially identified for improving DRS uptake (74 printable and 22 non-printable). Sixty-three sources were reviewed for adaptation after exclusion of material based on the relevance (Additional file 1, Table 2), and a final selection of 33 resources were then reviewed for adaptation to the local context. A total of 16 key themes were identified, which needed to be addressed in the adaptation and development of HEI (see Table 4).

**Table 4:** Main themes and source of information for development of the HE material development

Theme/ Subtheme and Source of Information	Illustrative Quotations	Implication for Development of HE Material
1. Main domain- Individual level-personal factors		
Knowledge, expectations and attitude		
1.1 Lack of knowledge on DR &DRS		
*SIs* ^*a*^ -Lack of biological knowledge of the eye, DR affects the back of the inside of the eye and changes are not visible from outside. -Lack of understanding of early asymptomatic stage. *PWs* ^*b*^ -Necessity of providing distinct information to make PwDM aware of DR.	*“We don’t know about eye issues that can occur with diabetes, hospital staff need to make us aware about that” [PwDM_PW]* *“We don’t know about DR blindness or that effects of diabetes on the eye leading to blindness” [PwDM_PW]*	-Inclusion of information on DM caused by high sugar levels in blood, this will lead to changes of blood vessels at the back of the inside of the eye which are not visible from outside. -Incorporation of graphics and animations to explain the changes in the eye.
1.2 Lack of knowledge on referral system		
*FGDs and SSIs-* ^*c, d*^ *-*Lack of clear information on referral processes (where to go, when to go, how to access, etc.). -Inadequate information in the referral letter. *PWs-* -Need of clear stepwise guide on directions of reaching to eye clinic from the medical clinic, with a suggestion to include a map and how to get an appointment. -Information on procedures that will take place at the eye clinic, days and time of eye clinics. -Forgetfulness of the information relevant to the eye screening appointment. *SIs-* -Suggest including a flow chart about the process of referral pathways – step wise actions to go from the medical clinic to eye clinic.	*“We forget what doctor said when come out form the doctor’s room. Sometime people don’t like to ask again from doctor, thinking that doctor will blame”* *[PwDM_PW]* *“At the very first time we do not aware, don’t we? One will say this way, other will say that way only wasting of time” [PwDM_PW]* *“List out the availability of eye clinics that diabetic patients can attend” [Ophthalmologist_SI]*	-Inclusion of information on availability of free services at the nearest eye clinic. -Map with directions (in the leaflet), how to get an appointment of out-patient eye clinic, the details of eye examination/consultation processes happen at each stage. -Provide a space in the leaflet to mention details of next appointment (to be documented by the eye doctor). -Inclusion of a flowchart guidance on processes at eye clinic/eye hospital.
1.3 Attitude on uptake of DR services		
*SIs* -Need to emphasise the necessity of DRS even without having visual symptoms. *PW-* Reluctant to uptake services due to long waiting time at the eye clinic.	*“Emphasise that diabetic retinopathy changes are not visible to outside therefore you won’t be aware about this problem until you become blind” [Media personnel_SI]* *“some time whole day we wait in the queue, but no treatment given”* *[PwDM_PW]*	-Emphasise on early asymptomatic phase and need of regular screening even without any symptoms.
1.4 Attitude of lack of perceived threat on DR blindness		
*SIs* -Benefits of action (screening) and threats of inaction (sight loss). *PWs* -Benefits of annual screening, DR assessment and treatment at the eye clinic.	*“People don’t know about DR, we think it as a just eye check-up, we don’t know that it is important to check eyes” [PwDM_PW]*	-Highlight the danger of losing sight due to DM / DR and it is irreversible. -Information on early screening, detection and treatment can prevent sight loss.
1.5 Attitude of fear of uptake of services		
*FGDs -* -Fear of dilated fundoscopy, -Need of accompaniment following dilatation, -Lack of knowledge on process and requirement of pupil dilatation in retinal examination. *SSIs* -PwDM reluctant to undergo pupil dilatation *PWs* -Ensure details of eye examination do not promote fear. *SIs* *-*Recognise the discomfort side effects but place emphasis in the benefits of the eye examination -Fear to uptake laser and surgery.	*“There is a drop before eye examination, and putting it to the eye is very painful” [PwDM_PW]* *“It was like burning, and covered the vision like fog” [PwDM_PW]* *“My eyes became blue, it was such an electric shock.” [PwDM_PW]* *“bringing a guardian is compulsory for putting eye drop, otherwise you can’t move due to blurred vision” [PwDM_PW]* *“Mention that they have to undergo dilated fundal examination to examine the inside of eye” [Optometrist_SI]*	-Inclusion of information on why there is a need for pupil dilatation (to have a better view of the back of the inside of the eye). Provisions of reassurance by an expert patient -Include information on blurring as a temporary side effect but include reassurance that this is normal. -To include guidance that accompaniment needed. -Guidance that no driving recommended following examination for up to 4–6 h time period.
1.6 Current level of expectation		
*SIs* -Need to describe DR as a separate entity, and it is different from cataract, glaucoma and vision problems that would require spectacles *PWs* -Confusions on DR screening over other forms of eye examination (refraction and cataract assessment)	*“We don’t know about the diabetic retinopathy and how it could be treated, we though cataract surgery and spectacles is the solution” [PwDM_PW]*	-Inclusion of information DR as a separate eye problem and undergoing cataract surgery and using spectacles will not correct all visual problems. - Need of salient information on DR and DRS.
1.7 Expectation of Information on outcome of eye examination		
*SIs* -Describe the outcomes of screening	*“Patients tend not to come once they have undergone a few treatment sessions, therefore need to tell the importance of attending for treatment regularly” [Consultant Ophthalmologist_PW]*	-Include information on outcome of the DRS and necessity of undergoing treatment as required. -Inclusion of information on availability of free DR treatment facilities at the eye clinic/public sector hospital.
2.Main domain - Environment		
Social norms and access to information		
2.1 Social norms in the local context (lay referral systems)		
*FGD* -Practice of indigenous medicine, engage in religious activities and use of home remedies, -Belief of blindness occur due to ageing, karma or faith. *PW* -Decision making for women happen at the home environment decided by a male member of family.	*“Doing ‘Bodhi puja’ activities and some other ‘bali-thowil’ (rituals and religious activities)” [PwDM_FGD]* *“Keep tea powder on the eye, washing eye using pomegranate leaves and jasmine” [PwDM_DM]* *“With aging diabetic is a normal disease. Also, most of these diseases occur due to our own sins” [PwDM_DM]*	-Provision of information to refrain from those activities.
2.2 Access to information and influences from the environment		
*SSI and SIs* -Lack of availability of health educational interventions on DR in local languages. *PW* -Difficulties in communication with the providers (language barriers and usage of technical terms).	*“We don’t know about eye issues that can occur with diabetes, hospital staff need to make us aware about that” [PwDM_DM]* *“We do not have proper methods on health education especially for diabetic retinopathy” [Medical officer_SSI]*	The need of HEI in local languages.
3. Main domain -Mode of Delivery		
Medium, personnel and place of delivery		
3.1 Views on medium of delivery		
*SIs* -Video, leaflet and poster as the suitable media for this context. *PWs* -Majority preferred a leaflet, -Majority of the participants who speak Tamil preferred a video-based health educational intervention (assessed using a ranking system at PW).	*“Video, leaflet and booklet are the preferable medium. We can have a video for about 15 min. Or quick advert < 1 min.” [Optometrist_SI]*	-Investigators consensus - Development of a leaflet and a video intervention in local languages (original version in English)
3.2 Views on place of delivery		
*SIs* -Medical clinic as the best place to deliver. *PWs* -Majority wanted HE to be conducted at the medical clinics.	*“Medical clinic is the best place to deliver this education intervention” [Expert PwDM_SI]*	-Field testing of the HEI at medical clinic.
3.3 Views on personnel of delivery		
*SIs* -Delivery by doctor or a nurse. *PWs* -Health education should be done by a doctor or a nurse, best delivered by a doctor.	*“Health education can be delivered verbally to a small group by a doctor, or need an educator to deliver information in especially in Tamil” [Medical officer_SI]*	-Field testing delivery of the HEI by the physicians at the medical clinic
4. Main domain – Comprehensibility and Readability		
Comprehension, readability and terminology		
4.1 Difficulties in finding the terminology in local languages		
*PWs* -Difficulties in understanding the terms of; retina, diabetic retinopathy, laser, pupil, pupil dilation, blood glucose.	*“For dilating drops, dilatation of pupils, retina, retinopathy, use simple terms. Comprehensive eye examination word is hard to understand, mention that it is an examination of the back of the eye.* *To describe the word retina: use - inside of the eyes or wall of the eye at the back or describe retina is like the roll of a film camera”. [Optometrist_SI]*	-Use of phrases in local languages when there were no appropriate terms in local language.
4.2 Views on layout and format (printed material and video).		
*SIs* -Inclusion of information on question and answer format. -Incorporation of graphics and animations to explain that DR affects back of the inside of the eye. -Reduce the number of sentences per page. *PWs* -Usage of high-resolution images. -Usage of large fronts and large page sizes.	*“Use more images to get the attention” [Consultant Ophthalmologist_SI]* *“In the leaflet, each page can be divided using sub-topics, page 1-Title with a theme, 2nd – some background details of diabetes in Sri Lanka, 3rd – changes in the retina due to DM, 4th – screening and treatment options of DR, 5th – How to control DM, 6th – main messages, where to go for eye checking etc” [Community Physician_SI]*	-Follow the suggestions given
4.3 Usage of appropriate language matching the literacy level of the PwDM in the context		
*PWs* -Minimise the usage of technical terms and direct use of words in English. -Availability of the alternatives for illiterate PwDM. - Need of locally acceptable terminology to deliver information. *SIs* -Minimal usage of technical terms and usage of phrases when it is difficult to find the terms in local languages.	*“doctors explain fast and sometime can’t understand, they use English words in between which we cannot understand. We do not ask those back again due to fear that doctor get angry” [PwDM_PW]*	-Followed the suggestions given in development of HEI.
5. Main domain - Behaviour		
Skills of acquiring information and cues for action		
5.1 Component of potential behaviour change		
*SIs* -Sharing the experiences of PwDM, those who had STDR / acute loss of vision. PW - Skills and practice of acquiring knowledge and uptake of services	*“We can include a video clip of a patient who lost vision due to diabetic retinopathy telling her/his experience, by this ask diabetic people to go for annual Dr screening”* *[Lecturer in Media Communications_SI]*	-Inclusion of a video segment of a patient sharing the experiences of acute vision loss (e.g. vitreous haemorrhage)

^a^stakeholder interviews

^b^participatory workshops with PwDM

^c^focus group discussions with PwDM

^d^semi-structured interviews with providers

Thirty-three PwDM (20 Sinhala speaking, 13 Tamil speaking) attended participatory workshops. Overall there were low levels of knowledge of DR and DRS, a lack of perceived threat of DR blindness, combined with DR not being seen as a life-threatening condition, in particular during the asymptomatic phase. Commonly, for example, cataract surgery or the provisions of spectacles were understood to be the solution for all eye health problems. The use of medical terms, often in English, was also identified as a barrier to their understanding, including terms such as ‘retina’ and ‘pupil dilation’, although these terms could not be easily translated into the local languages. Knowledge about treatment options in government hospitals was also limited, combined with poor understanding of the referral pathways, and of the need for regular screening. The confusion about referrals and forgetfulness about appointments was often exacerbated by the lack of a printed referral form, and/or lack of information available in the local languages. Another barrier to uptake of services was negative attitudes about very long waiting times at clinics which deterred patients from regular screening. Fear about the procedures, such as pupil dilation, was another barrier.

In terms of environmental factors, social norms emerged as a key theme, which influence management of DM. Some PwDM practiced indigenous medication, whilst a common belief was also that blindness was an inevitable part of ageing, karma or faith which was not avoidable and therefore difficult to treat. The decision making on accessing services and requirements of an escort, especially for women, mostly happened at the family level, in what is a patriarchal society system. Therefore, development of HEI needed to be family-centred and any materials to be developed in a way that it would be easy to share.

In terms of readability of the HEI a common request was for clear and colourful images and to use large page size and font size for printable material. Stakeholders identified that an animated video could enrich understanding of biological changes to the eye. In terms of the mode of delivery there was an overall preference from the Tamil speaking groups for the video, whilst in contrast the Sinhala groups in general expressed a preference for printed reading material. All groups agreed that the medical clinic provided a useful space to deliver this HEI, whilst waiting for appointments.

### Field testing of acceptability, relevance and understanding

Overall as a result of the field testing of the video and leaflet, there were a number of lessons learnt about the relevance and acceptability of the HEI, reflected in 3 major themes; 1) levels of comprehension and readability, 2) actionability of uptake of services and 3) views on mode of delivery. The details of themes and sub-themes are available in Table 5.

**Table 5:** Main and sub-themes of field testing of the developed HEI

Main Domain	Theme	Sub-theme	Example Quotation
1) Comprehension and readability	1.1) Intelligibility of the leaflet and video	Understanding of diabetes lead to blindness and eye check-up prevent sight loss	*“Diabetes can cause a huge damage to the eyes. It can lead to blindness. We can spend little time and get our eyes checked and prevent this damage.” [HE_S22_64yrs_F]*
		Difficulties in reading the leaflet by some PwDM	*“I like the video because I can see it clearly. To read the leaflet I have to put some effort. It was bit of a hard work for me.” [HE_S03_65yrs_F]*
	1.2) Difficulty in interpreting figures and medical images	Difficulty in understanding of the fundus images (in page number 03-leaflet).	*“I could understand most of the things; However, I could not get the message from the pictures in the 2nd or the 3rd page. I cannot understand what is explained here.” [HE_M14_51yrs_M]*
	1.3) Level of simplicity and cultural appropriateness of the language style	Not preferring different colloquial languages in Tamil	*“This is Jaffna Tamil. It is difficult to follow the video.” [HE_M17 _65yrs_F]*
2)Actionability	2.1) Ability to extract key messages of referral uptake	Understanding importance of follow-up as a key message	*“I think the more serious message I captured from the video is that the ‘right follow up’ is very important to protect the sight’. Old lady’s story was interesting for me.” [HE_S21_58yrs_M]*
		Understanding of Facilities are available at XX hospital.	*“XX hospital is more capable of providing the latest treatments. We should get the maximum benefits out of it as diabetic patients.” [HE_S11_62yrs_M]*
3) Mode of Delivery	3.1) Preference over delivery at the medical clinic	Preference of delivering and effective use of waiting time at the medical clinic.	*“It is good to get the details like this at the Room 26 (medical clinic). After giving the leaflet I had enough time to read it, till I get my turn. I was sitting more than one hour.” [HE_T06_51yrs_M]*
	3.2) Usability and willingness to share the HE material	Level of sharing resources	*“My husband comes home late after work. He is tired after working and I am reluctant to discuss about my diseases when he is back home.” [HE_S27_50yrs_F]*
	3.3) Overall high social acceptability and attractiveness of the HEI	High acceptance of the delivered leaflet and video.	*“I prefer both leaflet and video, but for more common use, leaflet would be better. It is easy to carry inside my bag.” [HE_S06_71yrs_F]*

### Comprehension and readability

Overall the majority of the participants in both language groups stated that key messages were clear in both media of delivery. However, a common difficulty was interpreting some of the biological images, such as fundus images depicting the progression of DR and how vision varies. There were also difficulties in interpreting and understanding the relevance of numbers and percentages that described the disease burden of DR. PwDM also requested more information on the dangers of DR in the leaflet.

In terms of the cultural appropriateness of the language, the majority of the Sinhala patients were satisfied with the use of language in the video and leaflet. Whilst in contrast, some phrases and terms were in a different regional language for the Tamil group, and therefore more difficult to follow, as one 65 year old female explained, *“This is Jaffna Tamil, so it is difficult to follow the video”.* The Moor PwDM highlighted the limited representation of Moors in the video and were critical of the unintentional background footage of images of Tamil religious symbolism. A major theme across most patients and providers was the need to interact with someone about the video or leaflet to aid clarification. This was illustrated by a 65-year woman who explained that *“I didn’t understand the animations straight away. But after several explanations I could understand some of it”.*

The providers as well as the PwDM, suggested that lay-out and design of the leaflet and showing the directions to the eye clinic could be improved further; suggestions included incorporation of segments of the eye care hospital into the video.

### Actionability- understanding of referral processes

Overall there was good understanding of the key steps to undertake for referrals, including the need for regular screening and follow up, and of facilities available in the public sector eye hospitals: *“I think the more serious message I captured from the video is that the ‘right follow up’ is very important to protect the sight. The old lady’s story was interesting for me”.* Participants also showed an indicative positive attitude about intention to seek care. However, improved maps of the location of services were a common request.

### Mode of delivery

In terms of mode of delivery, the majority of the participants reflected on the usefulness of receiving the leaflet whilst waiting for the consultation. PwDM said they liked delivery of leaflet and video to be facilitated by their physician, so that it could support an interactive session. Yet at the same time a common complaint was that the environment was too noisy for the video: “*sounds and voices were not clear in some parts, because of the noisy environment inside the clinic”.* Providers also highlighted the practical difficulties in delivering the video at the clinic setting when there were no facilities for a larger number of PwDM.

A central component of the HEI was that it would prompt a willingness to share the resource with other family members. However, the field testing showed that in practise, there was limited showing of the video, and it was unclear the extent to which the leaflet was shared. The main reasons given were lack of any facility to watch and/or lack of time for a family member to watch a video. Overall there was a small preference for the leaflet in the field testing, because of the practical ease of carrying it and being able to refer to it.

Both PwDM and providers suggested that lay-out and design of the leaflet and showing the directions to the eye clinic could be improved further; suggestions included incorporation of segments of the eye care hospital into the video.

### Providers’ views on the delivery of HEI

The physicians who delivered the HEI stated that major practical issue was lack of time to deliver and talk through the material. One physician expressed her view as follows. *“A clinical setting at a hospital is very congested and busy. A Few doctors are there to provide for the needs of hundreds of patients. A single patient has a very limited time to spend with the doctor, not enough time for an efficient health educational intervention”*. Providers emphasised that unavailability of proper technical facilities and limited space to display the video was the main challenge they faced in delivering the HEI at the medical clinic. *“The video has a more likelihood of capturing people’s attention, but there must be proper tools to deliver it. There must be enough space and facilities for all this. In my opinion, the leaflet is far more practical when it comes to congested hospital setting.”*.

We observed the value of bringing the providers and other stakeholders along the journey from early consultation through to the final field testing. This was helpful to build their ownership to the developed material with a greater likelihood of utilising it as a HEI in future.

## Discussion

This study showed that the process of adapting HE material is not simply translation into the appropriate language. Instead adopting a participatory process, and working with PwDM and with providers, we identified important content and process issues, necessary to make the material relevant and acceptable, with some lessons learnt for scaling up. The key findings were, PwDM had low levels of knowledge and awareness on DRS, referral pathways and follow ups. Stakeholders prioritised the importance of using of simple language and need of highlighting the importance of DRS in asymptomatic phase. In the field testing we found that most of the PwDM could comprehend the content with overall good understanding of the key steps, except on a few occasions among Tamil and Moor ethnic groups. Additionally, we observed that there were technical and human resources constraints to deliver the HEI at a medical clinic setting. Our HE video and leaflet had satisfactory level of acceptability to the PwDM based on their verbal responses. This HEI would have a greater potential of using as an HEI to improve the referral uptake following further refinement according to the contextual requirements.

The World Health Organization (WHO) Shanghai Declaration stated that health promotion should be an essential component in all health systems to have an equitable access [26] and eye health promotion consists of a mix of HE, services improvement and advocacy [19]. In terms of DRS services uptake of screening and treatment is low, therefore an effective HE strategy is one essential component to ensure equitable access [19]. This is one of the first studies to describe the process of adaptation, development and assessment of an acceptable HEI on DR in Sri Lanka.

The use of participatory methods targeted at the intended community has been shown in a study to be an effective way to develop material which has a high level of acceptability [27]. This method enabled us to tailor the material to be more culturally sensitive, for example in terms of understanding any particular needs of different ethnic groups. The importance of culture sensitive adaptation of the material compared to the conventional translated material has been described in a controlled study conducted in a high income country migrant populations and showed that adapted material were significantly more useful than translated versions [28]. A study on development of HE material in hypertension for an Indo-Asian community stated that active participation of the targeted community was helpful in developing acceptable material [27]. We identified that the existing resources on DR were developed according to the various contextual requirements in different contexts.

Our study showed that overall knowledge on DM and DRS was low, and this needed to be addressed with the HEI. A study conducted in South India, in a population similar to Sri Lanka showed that only about 40.7% of PwDM had good knowledge on DM and DR and 9.6% has undergone DRS [29]. In addition, we identified various attitudinal and behaviour patterns such as fear, lack of understanding between DRS and routine eye check-up affects uptake. Similar findings were reported in other studies on barriers to uptake DRS [30, 31]. In addition, we observed that PwDM were reluctant follow up with screening and treatment when there was no threat to sight or during asymptomatic stage. A similar challenge was described in a systematic review of HE interventions to improve the adherence to glaucoma medication [32].

The field-testing phase showed that despite high level of literacy in Sri Lanka [33], the functional health literacy was low among some participants, and in particular there was difficulty in interpreting and understanding medical jargon and biological pictures. The WHO highlights that HE is necessary to improve functional health literacy, which in turn would reduce inequalities to access [18]. A systematic review on assessment of readability of ophthalmology HE material showed that most of the eye care HE material required high level of literacy for understanding [34]. Therefore, any HEI needs to be further developed in a way that is understandable to wide range of PwDM with various levels of functional health literacy. Another key element identified in our study was necessity of using locally derived, simple, and understandable terms (i.e., retina, diabetic retinopathy, pupil dilatation, vitrectomy etc.) in different dialects, as described in a study on glaucoma HE in Nigeria [35]. This shows that working on multiple languages requires a lot of competency in translation and back-translation, to maintain the integrity of the HEI.

Our study showed that socio-cultural adaptation of the material is a crucial factor to improve uptake, in a patriarchal society like in Sri Lanka. A study from Sri Lanka showed that PwDM behave with regard to DM based on various socio-cultural beliefs [36]. Therefore, gender and culture sensitive HE strategies should be developed, where females are mostly not involved in decision making [37]. A similar finding of the importance of family in making decisions and gender role of women on service uptake has been described in a cataract surgery uptake study done in Tanzania [38]. We therefore developed the HEI in a way that it could be shared with family members, although in practise there was little evidence of sharing.

Our study showed an overall satisfactory level of acceptability for the leaflet and video material. We showed and expressed preference among the participants about the use of a video during the participatory development phase. A systematic review of assessing the efficacy of video-based HE to modify behaviour in handling health related issues showed that videos showing actual people can be more effective than graphical presentation [39]. However, the field-testing phase indicated that video used in the clinic was problematic for several reasons. It might be that finding an alternative way to present the video, for example using a smart phone / social media might be a more pragmatic approach which also offers wider coverage, as piloted in a study conducted in a similar community in South India for stroke survivor education [40]. We identified that either of the material may be insufficient as a stand-alone HEI and the video and the leaflet should both be more complementary to each other.

One of the key findings in our study was that HE benefits from a more interactive approach where there is an opportunity to discuss the material with a provider and seek clarification. The clinic work load [41], lack of prioritising the HE at clinic setting [42] and lack of training of human resources [43] have been described as barriers to HE in other studies. In our study the lack of physician time was identified as major barrier and highlighting the need to consider task sharing or shifting and finding other staff or expert patients who might play an important educator role. The need of a health educator for a busy clinic has been suggested in health promotion study done in USA on Glaucoma [44].

### Limitations

One limitation of the study was that, we assessed the acceptability of the HEI using subjective responses of the PwDM and providers. We did not assess knowledge and awareness of the PwDM at the baseline. Our study sample consisted mostly of elderly PwDM. Further, the small sample size may not represent the diversity of the community in this region. Therefore, conclusions drawn from this study may not generalisable. We did not assess the variations in acceptability by age, gender or socio-economic status.

## Conclusions

The development of HE material in DR is a complex process, requires adaptation and development suitable to the local context. The process of adapting the health educational material is not simply translation into the appropriate language but rather an individualised tailored approach in different health services to meet the needs of variuos patient communities. Socio-cultural norms should be considered when defining the actionable steps. We can conclude that there is a satisfactory level of acceptability for this HEI and to deliver at a medical clinic setting would require further development of human resources and infrastructure appropriate to the intervention.

## Recommendations

- ▪ Improve functional health literacy by further simplification of the resource, including minimal use of medical jargon.
- ▪ Strengthen the interactive use of HEI, with a skilled educator to discuss, clarify and counsel.
- ▪ Explore options for task sharing or task shifting the educator role from the physician to another staff member and or expert person with DM.
- ▪ Use the waiting time at the medical clinic as a dedicated and targeted time for HE. This should include ensuring there is adequate space, including a quiet space for delivery of the video material.
- ▪ Develop the video into shorter film clips for use at the waiting areas of the medical clinics before consultation, prompting to clarify the queries during consultation to improve access.
- ▪ Consider options for developing a cadre of expert patients who could work in local dialects and may be in better position to work with minor ethnic groups, and to engage with other family members.
- ▪ This HEI should be one component of a wider health promotional strategy to improve the uptake of DRS in Sri Lanka. A next step is then to test the effectiveness of this strategy in a controlled trial.

## Additional files

Additional file 1: Search on health educational interventions / material on improving the referral uptake. (DOCX 16 kb)

Additional file 2: 2.1: Leaflet health educational intervention in English and local languages (Sinhala andTamil) and 2.2: Outline and script in English and local languages (Sinhala and Tamil) of the video healtheducational Intervention. (ZIP 3663 kb)

Additional file 3: Topic guides for field testing of the HEI – for service users and service providers. (DOCX 18 kb)

## Abbreviations

DM: Diabetes mellitus
DR: Diabetic retinopathy
DRS: Diabetic retinopathy screening
HE: Health education
HEI: Health educational intervention
HIC: High income countries
PEMAT: Patient Education Material Assessment Tool
PwDM: People with diabetes mellitus
SCT: Social Cognitive Theory
WHO: World Health Organization
