# Supplementary material for: Process of adaptation, development and assessment of acceptability of a health educational intervention to improve referral uptake by people with diabetes in Sri Lanka
Source: BMC Public Health. 2019 May 21;19:614. doi: 10.1186/s12889-019-6880-4 (PMC6528317; doi:10.1186/s12889-019-6880-4)
Supplement: Supplementary file 1 — Search on health educational interventions / material on improving the referral uptake. (DOCX 16 kb) [file 12889_2019_6880_MOESM1_ESM.docx]

**Additional File 1 - Search on health educational interventions / material on improving the referral uptake**

**Table 1. Search Terms**

| Terms for search of material –  1 Diabetes Mellitus  2 Diabetes Complications  3 Diabetic Retinopathy  4 Health Education  5 Patient Education  6 Health Promotion  7 Diabetic Retinopathy Screening  8 Diabetic Retinopathy Blindness | Main sources of the adapted material –  -National Health Services – United Kingdom.  -University of Melbourne – Australia  -Diabetic Eye Screening Program – Northern Ireland  -National Eye Health Education Program – United States  -Diabetes UK  -National Health Services – Scotland  -Diabetic Retinopathy Screening Program for Aboriginal People – Australia  -Public Health Agency – Canada  -Royal National Institute of Blindness – United Kingdom  -Health Promotion Board – Ministry of Health – Singapore  -Moorfields Eye Hospital – United Kingdom  -Queen Elizabeth Hospital – Birmingham – United Kingdom  -National Eye Institute – United States  -Vision Initiative – Victoria – Australia  -Department of Health – Australia  -International Diabetes Federation  -Retina Group – Washington – United States  -National Library of Medicine – United States  -The Eye Centre – Video Library  -Diabetes UK – Learning Zone  -Medline Plus – Video Archives |
| --- | --- |

**Table 2. Summary of the electronic search of HE material on DR and DRS.**

| **Type** | **Total number of items retrieved in search** | **DM** | **DR** | **DRS** | **DR Rx** | **DR All** | **Number of items eligible in adaptation** | **Number of items with high PEMAT Score**  [>50% of the score] | **Number of items used in adaptation** |
| --- | --- | --- | --- | --- | --- | --- | --- | --- | --- |
| Poster | 28 | 16 | -- | 12 | -- | -- | 18 | 09 | 01 |
| Leaflet & brochure | 33 | 03 | 13 | 13 | 04 | -- | 29 | 17 Brochures 12 Leaflets | 12 |
| Videos | 24 | 02 | 11 | 05 | 06 | -- | 22 | 13 | 9 |
| Tip sheets | 07 | 02 | 04 | -- | 01 | -- | 04 | 04 | 4 |
| Info graphics & flipcharts | 31 | 09 | 10 | 07 | 05 | -- | 22 | 12 | 7 |
| Information Articles | 81 | 24 | 41 | 14 | 02 |  | -- | N/A | - |
| Banner | 10 | -- | -- | 10 | -- |  | 01 | 01 | - |
| Web based education | 216 | 32 | 59 | 12 | 11 | 102 | -- | N/A | - |
| Resource & ideas | 13 | 04 | 02 | 01 | -- | 06 | -- | N/A | - |
| Audio | 04 | -- | 01 | 03 | -- | -- | -- | N/A | - |
| Software applications (Apps) | 02 | 02 | -- | -- | -- | -- | -- | N/A | - |
|  | 449 |  |  |  |  |  | 96 | 68 | 33 |
