# Supplementary material for: Process of adaptation, development and assessment of acceptability of a health educational intervention to improve referral uptake by people with diabetes in Sri Lanka
Source: BMC Public Health. 2019 May 21;19:614. doi: 10.1186/s12889-019-6880-4 (PMC6528317; doi:10.1186/s12889-019-6880-4)
Supplement: Supplementary file 2 — 2.1: Leaflet health educational intervention in English and local languages (Sinhala andTamil) and 2.2: Outline and script in English and local languages (Sinhala and Tamil) of the video healtheducational Intervention. (ZIP 3663 kb) [file 12889_2019_6880_MOESM2_ESM.zip › 5_BMC_HEI_Additional File 2.1_Leaflet_R2.pdf]

Additional File 2 - Leaflet Health Educational Intervention in English and local languages (Sinhala and Tamil)

High Blood Sugar Can Harm Eyesight

Your Guide to Prevention of Visual Impairment and Blindness due to Diabetic Eye Ailment

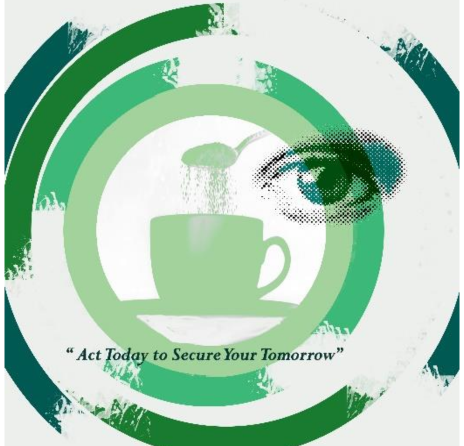

“ Act Today to Secure Your Tomorrow”

Health promotional material developed as part of a feasibility study in developing and diabetic retinopathy screening programme in Sri Lanka.

Copyright © 2018 by Dr. M.M.P.N Piyasena All rights reserved. This leaflet or any portion thereof maynot be reproduced or used in any manner whatsoever without the express writen permission of the author .

Are you aware that diabetes can cause visual loss?

In Sri Lanka for every 100 adults (above 20 years), 20 have diabetes

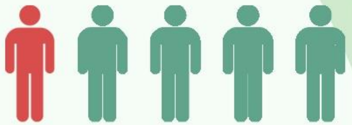

Substantial number of these people’s eyes are affected and cause visual loss.

For every three people with diabetes one can have diabetic eye ailment,

you could be one of them.

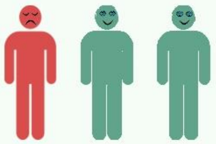

Copyright © 2018 by Dr. M.M.P.N Piyasena All rights reserved.

What is diabetic eye ailment?

- High sugar levels in the blood in diabetes leads to diabetic eye ailment.
- Diabetes affects the small blood tubes which supply the light sensitive layer at the back of the eye (which is called retina).
- These blood tubes starts bleeding in diabetes
- These tubes grow abnormally at the back of your eyes then bleed or leak, which you cannot see from outside.
- This can progress and lead to visual impairment and blindness if not identified and treated in time.
- This condition is known as “Diabetic Eye Ailment ”.

Copyright © 2018 by Dr. M.M.P.N Piyasena All rights reserved.

Different stages of diabetic eye ailment and how you would see

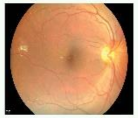

Normal back of the eye

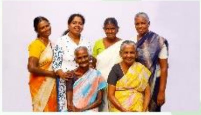

You have normal vision

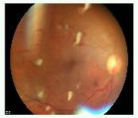

Back of the eye with moderate diabetic eye ailment

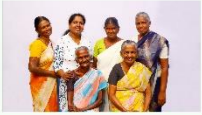

Still you have the normal vision

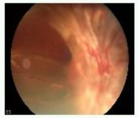

Back of the eye after developing severe diabetic eye ailment

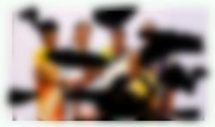

Your vision gets blurred only after having severe diabetic eye ailment

Copyright © 2018 by Dr. M.M.P.N Piyasena All rights reserved.

Copy Right Holder of the Leaflets - ©Dr.M.M.P.N.Piyasena-London School of Hygiene and Tropical Medicine (November/2018)

Can we prevent this condition by undergoing cataract surgery or using spectacles for reading or seeing people or objects from a distance?

- Diabetic eye changes should not be confused with cataract or spectacles.
- We use spectacles to correct defects in the outer most part of the eye or due to changes in the lens power.
- The cataract surgery and intra ocular lens implantation will be done when natural lens become opaque and thick.
- Therefore, usage of spectacles or undergoing cataract surgery would not prevent you from getting the diabetic eye changes.
- Further there are no “eye drops or local remedies” that prevent this condition.
- Because of that you will have to undergo diabetic eye examination even after undergoing cataract surgery or even if you are using spectacles (or even if you have any other eye disease/s).

Copyright © 2018 by Dr. M.M.P.N Piyasena All rights reserved.

Why is it important to check early and regularly for diabetic eye ailment ?

- Main feature of this condition is, you would not feel any symptoms related with diabetic eye ailment at the early stages.
- Checking eyes early, can detect changes at back of your eyes, before you are aware of them.
- If these changes are detected in time, treatment is very effective at preventing sight loss.
- It is therefore important to have your eyes checked regulary.

Copyright © 2018 by Dr. M.M.P.N Piyasena All rights reserved.

How would your eyes be checked at medical clinic ?

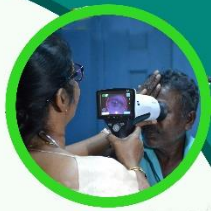

- Your doctor will examine your eyes using a special eye camera.

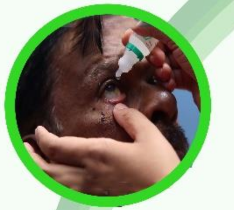

- Eye drops will be instilled to your eyes to have a better view of the back of your eyes.
- You should not worry about the difficulties you have after the drops, since these will become normal after weaning off the effect of drugs.

Copyright © 2018 by Dr. M.M.P.N Piyasena All rights reserved.

What you should do after eye examination at medical clinic?

- The outcome of eye examination will be informed by your doctor at the medical clinic.
- You will be referred to National Eye Hospital - Colombo eye clinic, if found to have diabetic eye ailment which requires further assessment/ investigation.

Or else:

- You will be asked to undergo same eye examination in one-year time, if there are no signs of diabetic eye ailment or minor signs which do not require further assesment at this stage.
- Please note that here we will examine only for diabetic eye ailment and you may present to any eye clinic if you have any other eye problem/s.

Copyright © 2018 by Dr. M.M.P.N Piyasena All rights reserved.

Copy Right Holder of the Leaflets - ©Dr.M.M.P.N.Piyasena-London School of Hygiene and Tropical Medicine (November/2018)

Steps to follow to go to eye clinic

- National eye hospital Colombo has all the advanced treatment and investigation facilities for diabetic eye ailment, at no cost!
- You will be treated by specialized eye doctors there.

Find the location of the eye clinic using folowing map

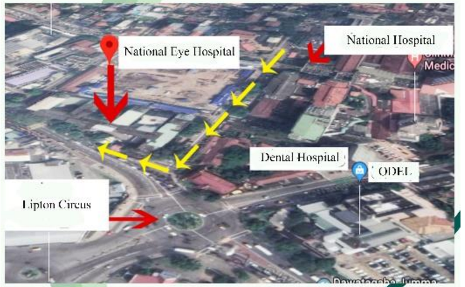

Copyright © 2018 by Dr. M.M.P.N Piyasena All rights reserved.

What you should do at eye clinic?

Enter in to National Eye Hospital through gate number 4 and go to the OPD number issuing counter.

There, you will have to obtain an OPD ticket by providing name, age and place of residence

Afterwards check your vision at room number 10.

After checking acuity you will be directed to the relevant eye clinic according to your OPD number

- Afterwards doctor will provide instructions of further steps and management plan.

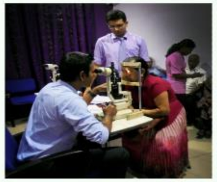

Copyright © 2018 by Dr. M.M.P.N Piyasena All rights reserved.

Please remember the followings when you come for your next visit

- Bring your pair of glasses (spectacles) and contact lenses you wear along with lens solution for contacts.
- You may asked to bring someone to escort you to the appointment.
- Eye drops may affect your vision for a few hours, so you should not drive/ ride bicycles/ trishaws after your appointment.
- You should ask and make clear about the next steps from your eye doctor before you leave the clinic.
- Please note down the next appointment date, time and room number back of this booklet.
- Please remember to bring clinic notes, previous eye examination reports when coming for the next visit.

Copyright © 2018 by Dr. M.M.P.N Piyasena All rights reserved.

Date	Hospital name	Clinic room number

Health promotional material developed as part of a feasibility study in developing and diabetic retinopathy screening programme in Sri Lanka

Copyright © [illegible] [illegible]

LONDON SCHOOL of HYGIENE & TROPICAL MEDICINE

Copy Right Holder of the Leaflets - ©Dr.M.M.P.N.Piyasena-London School of Hygiene and Tropical Medicine (November/2018)

Leaflet - Tamil medium

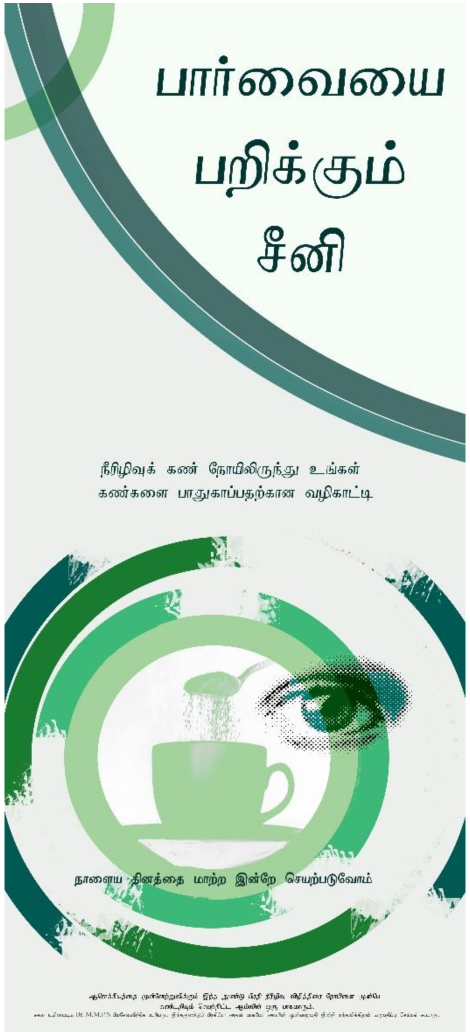

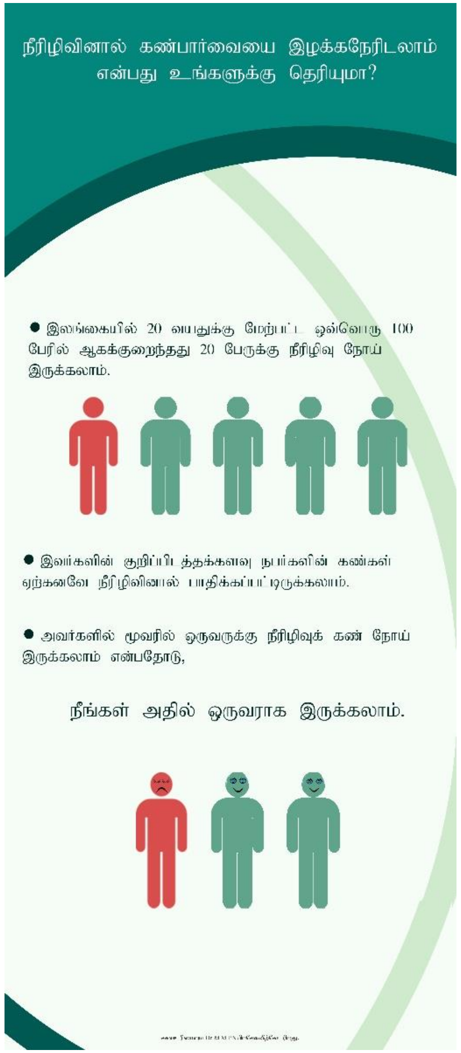

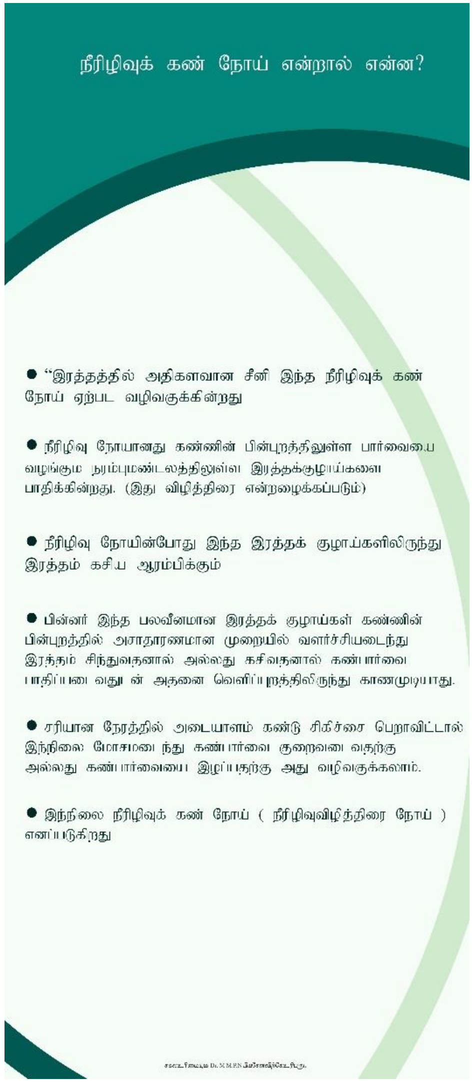

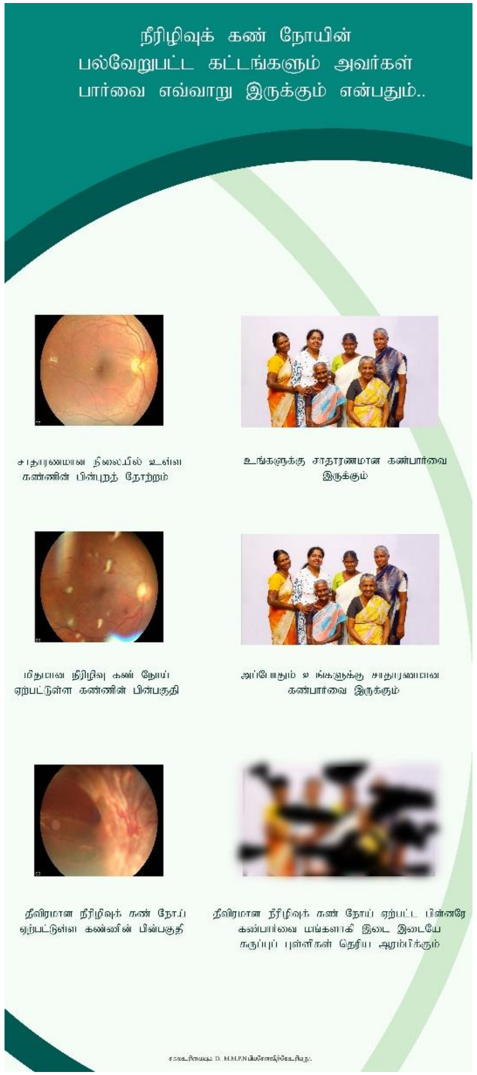

Copy Right Holder of the Leaflets - ©Dr.M.M.P.N.Piyasena-London School of Hygiene and Tropical Medicine (November/2018)

கட்ராக் அறுவை சிகிச்சை மூலம் அல்லது மூக்குக் கண்ணாடி பாவிப்பதன் மூலம் நீரிழிவுக் கண் நோயினை தடுக்க முடியுமா?

- நீரிழிவுக் கண் நோயை மூக்குக் கண்ணாடி மற்றும் கட்ராக் நோயுடன் குழப்பிக்கொள்ளவேண்டாம்

- நாம் கண்ணாடி பாவிப்பது கண்ணில் வெளிப்புறத்திலுள்ள (கருவிழி) குறைபாடுகளை திருத்திக்கொள்வதற்கு அல்லது வில்லையின் பார்வை ஆற்றலை மாற்றுவதற்கு ஆகும்.

- இயற்கையான எமது கண்வில்லை தடிப்பாகி ஒளி ஊடுருவமுடியாத நிலைக்கு வரும் பொழுது இந்த விழிவெண்படல சிகிச்சையும் கண்ணின் உள்ளே வில்லை பொருத்துவதும் மேற்கொள்ளப்படுகிறது.

- அதனால் வெண்படலம் அகற்றும் அறுவை சிகிச்சை செய்தாலும் மூக்குக் கண்ணாடி பயன்படுத்தினாலும் நீரிழிவு விழித்திரை நோய் ஏற்படுவதனை தடுக்க முடியாது.

- அவ்வாறே நீரிழிவு கண் நோயை சொட்டு மருந்துகள் மற்றும் கைமருந்துகள் மூலம் தடுத்துக் கொள்ள முடியாது.

- எனவே வெண்படலம் அகற்றும் அறுவை சிகிச்சை செய்தாலும் மூக்குக் கண்ணாடி பயன்படுத்தினாலும் (வேறு கண் நோய்களுக்கான சிகிச்சை பெற்றிருந்தாலும்) நீரிழிவு கண் நோயை கண்டு பிடிக்கும் பரிசோதனையை செய்தல் வேண்டும்.

நீரிழிவு விழித்திரை நோயினை கண்டுபிடிக்கும் பரிசோதனையை செய்துகொள்வது ஏன் முக்கியமானது?

- இந்த நோயின் ஆரம்பகட்டங்களில் அது தொடர்பான எந்தவொரு அறிகுறியினையும் காட்டாதிருப்பது அதன் பிரதான பண்பாகும்.

- நீங்கள் அந்த நோய் அறிகுறிகளை உணர்வதற்கு முன்னரே அதற்கான கண்டுபிடிப்பு பரிசோதனையின் மூலம் அதனை அடையாளம் கண்டுகொள்ளலாம்.

- இம்மாற்றங்களை முன்கூட்டியே அடையாளம் கண்டு சிகிச்சைபெற்றால் அது வெற்றியளிக்கலாம் என்பதோடு அதன் மூலம் பார்வை குறைவதனை அல்லது பார்வை இழப்பதனை தடுத்துக்கொள்ளலாம்.

- எனவே தொடர்ச்சியாக கண்களை பரிசோதனை செய்துகொள்வது முக்கியமானது.

மருத்துவ பரிசோதனையில் (கிளினிக்) உங்கள் கண்கள் எவ்வாறு பரிசோதிக்கப்படும்?

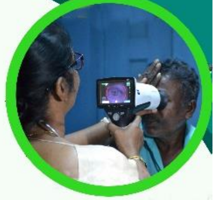

- உங்கள் வைத்தியர் விசேட கமரா ஒன்றின் மூலம் நீரிழிவுக் கண் நோய் இருக்கின்றதா என பரிசோதித்துப் பார்ப்பார்.

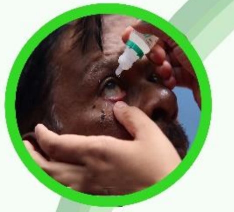

- கண்ணின் பின்பகுதியினை உட்பக்கத்தால் தெளிவாக பார்ப்பதற்கு விசேட சொட்டுமருந்து சில துளிகள் கண்ணில் விடப்படும்.

- துளிகள் கண்ணுக்கு விடப்பட்ட பின்னர் சிறிய அசௌகரியம் ஏற்பட்டாலும் அதன் செயற்பாடு சிலமணித்தியாலங்களில் குறைவடைவதனால் அது தொடர்பாக பயப்பட வேண்டியதில்லை.

நீங்கள் அடுத்து என்ன செய்வீர்கள்?

- கண் பரிசோதனை அறிக்கையை வைத்தியர் மருத்துவ சந்திப்பின்போது உங்களுக்கு அறிவிப்பார்.

- அவ்வாறு பரிசோதித்த பின் மேலதிக மதிப்பீடு செய்யப்பட வேண்டிய நிலையில் நீரிழிவு கண் நோய் அறிகுறிகள் இருந்தால் மேலதிக பரிசோதனைக்காக கொழும்பு கண் தேசிய வைத்தியசாலைக்கு செல்லுமாறு ஆலோசனை வழங்கப்படும்.

அல்லது

- அங்கு உங்களுக்கு நீரிழிவு கண் நோய் அறிகுறிகள் இல்லாதிருப்பின் அல்லது மேலதிக மதிப்பீடு தேவைப்படாத அறிகுறிகள் சிறியளவு இருப்பின் மீண்டும் ஒருவருடத்தின் பின்னர் இதே போன்றதொரு கண் பரிசோதனையைச் செய்யுமாறு கேட்டுக்கொள்ளப்படுவீர்.

- குறிப்பு - இங்கு நீரிழிவு கண் தொடர்பாக மாத்திரமே பரிசோதிக்கப்படும். உங்களுக்கு வேறு ஏதாவது கண் தொடர்பான பிரச்சினைகள் இருப்பின் வேறு கண் பரிசோதனைகளுக்கு செல்லவேண்டும் என்பதனை கருத்திற் கொள்ளவும்.

Copy Right Holder of the Leaflets - ©Dr.M.M.P.N.Piyasena-London School of Hygiene and Tropical Medicine (November/2018)

கண் பரிசோதனைக்கு செல்வதற்கு பின்பற்றவேண்டிய படிமுறைகள்

- தேசிய கண் வைத்தியசாலையில் நீரிழிவுக் கண் நோய்க்கான சகலவித முன்னேற்றமான பரிசோதனைகளுக்கும் சிகிச்சைகளுக்குமான வசதிகள் இலவசமாக செய்து கொடுக்கப்படுகின்றது.

- நீரிழிவுகண் நோய் தொடர்பாக தேர்ச்சிபெற்ற வைத்தியர்களால் அங்கு சிகிச்சையளிக்கப்படும்.

கண் மருத்துவ பரிசோதனை பிரிவிற்கு செல்வதற்கு பின்வரும் வரைபடத்தை பயன்படுத்தவும்

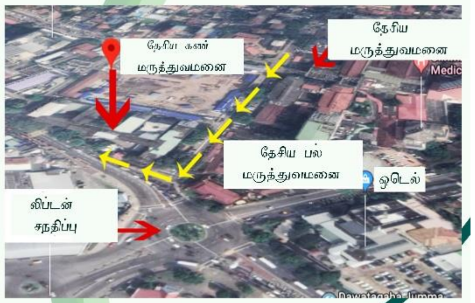

கண்களை பரிசோதித்த பின்னர் நீங்கள் என்ன செய்யவேண்டும்?

கொழும்பு, தேசிய கண் வைத்தியசாலையின் 4ம் இலக்க வாயிற் கதவினால் உள்ளே வந்து வெளி நோயாளருக்கான இலக்கம் வழங்கப்படும் விசாரணைக் கூடத்திற்கு செல்லவும்

அங்கு உங்களுடைய பெயர், வயது,விலாசம் போன்ற தகவல்களை வழங்கி வெளிநோயாளர் பிரிவின் மருத்துவ சீட்டைப் பெறவேண்டும்

பின்னர் 10ம் இலக்க அறையில் கண் பார்வையினை பரிசோதித்துக்-கொள்ளவும்

பார்வையை பரிசோதித்த பின்னர் உங்கள் வெளிநோயாளர் இலக்கத்தின்படி உரிய கண் மருத்துவ பரிசோதனைக்கு அனுப்பப்படுவீர்கள்.

- பின்னர் அடுத்தகட்ட செயற்பாடுகளுக்காகவும் திட்டமிடலுக்காகவும் கண் வைத்தியர் அறிவுறுத்தல்களை வழங்குவார்.

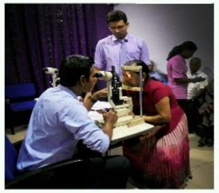

[illegible]

அடுத்த முறை வரும்போது பின்வருவனவற்றை நினைவில் வைத்துக்கொள்ளுங்கள்

- நீங்கள் அணியும் மூக்குக்கண்ணாடி, கண் வில்லை, மற்றும் அதற்காக பயன்படுத்தும் திரவம் என்பவற்றை எடுத்து வரவும்

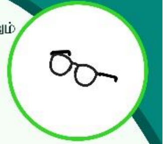

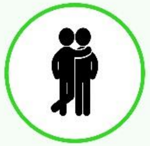

- அடுத்த சந்திப்பிற்கு வரும்போது இன்னொருவரையும் துணைக்கு அழைத்து வரும்படி கேட்கப்படலாம்

- கண் சொட்டுமருந்தினால் வைத்திய சந்திப்பின் பின்னரும் பார்வை சில மணிநேரம் பாதிக்கப்படலாம். எனவே வாகனம் செலுத்தவோ, மோட்டார் சைக்கிள், முச்சக்கரவண்டி ஓட்டவோ கூடாது.

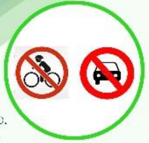

- நீங்கள் வைத்தியசாலையை விட்டு வெளியேறுவதற்கு முன்னர் அடுத்தகட்ட நடவடிக்கைகள் தொடர்பாக கண் வைத்தியரிடம் தெளிவாக கேட்டு தெரிந்து கொள்ளவேண்டும்.

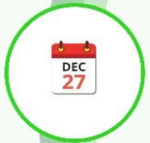

- இங்கு மீண்டும் வர வேண்டியதிகதி, நேரம் மற்றும் அறையின் இலக்கம் என்பவற்றை இதன் மறுபக்கத்தில் குறித்துக்கொள்ளவும்

- மீண்டும் கண் மருத்துவ பரிசோதனைக்கு வரும் போது உங்கள் மருத்துவகுறிப்பு,கடந்தமருத்துவ பரிசோதனை அறிக்கைகள் என்பவற்றை எடுத்துவரவும்

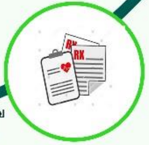

[illegible]

திகதி	வைத்தியசாலையின் பெயர்	அறை இலக்கம்

[illegible]

LONDON SCHOOL of HYGIENE & TROPICAL MEDICINE

Copy Right Holder of the Leaflets - ©Dr.M.M.P.N.Piyasena-London School of Hygiene and Tropical Medicine (November/2018)

Leaflet - Sinhala medium

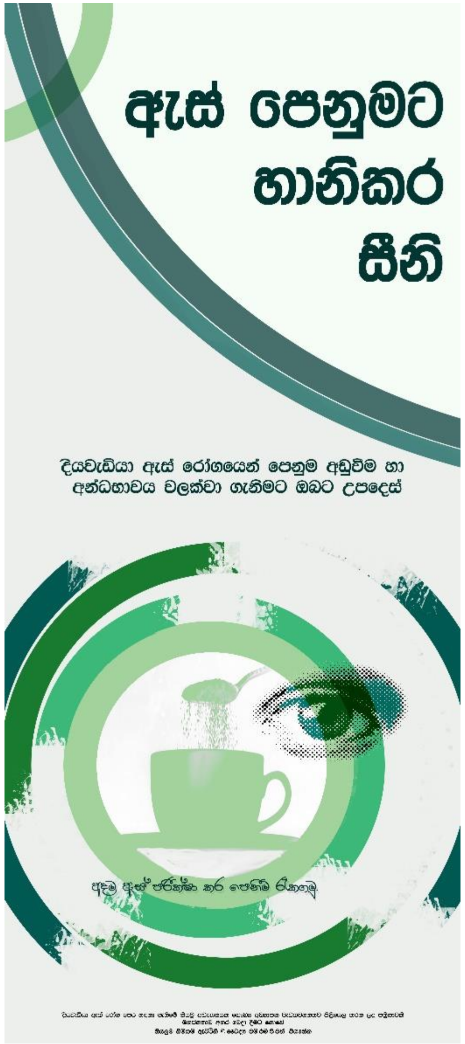

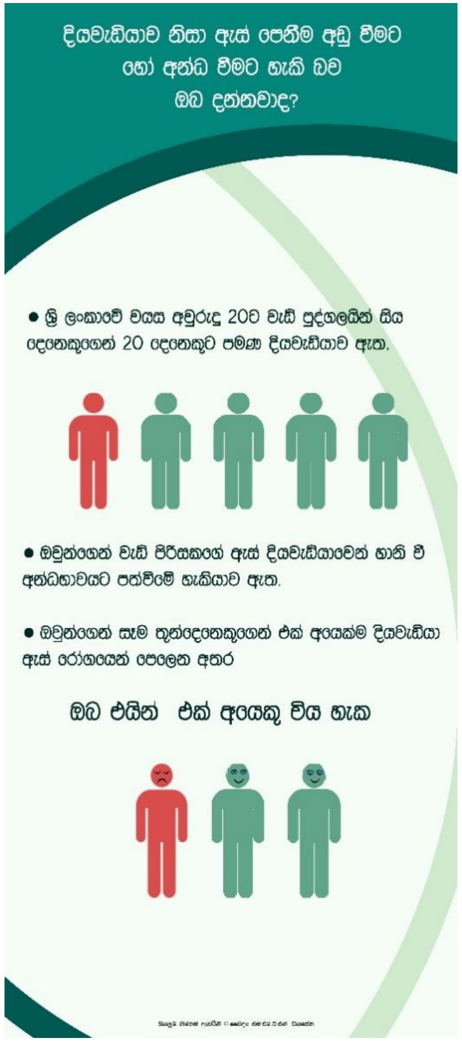

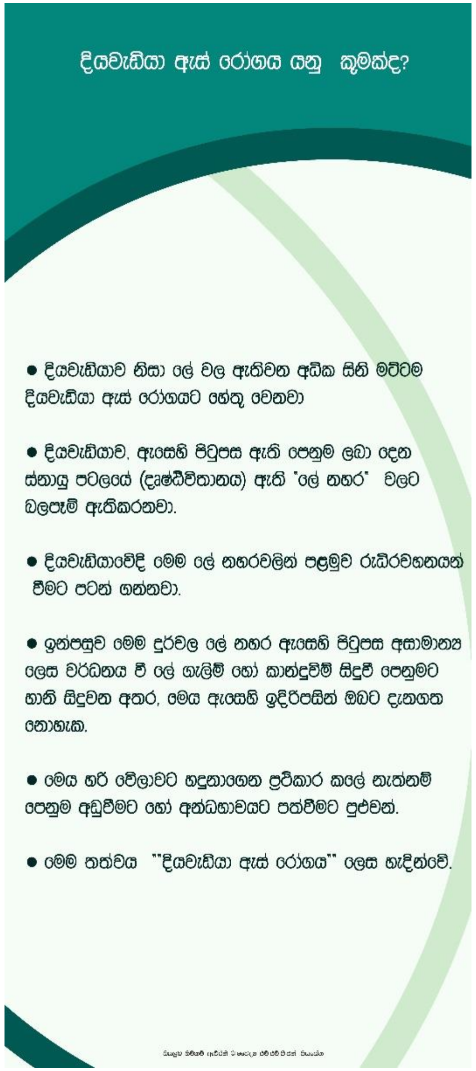

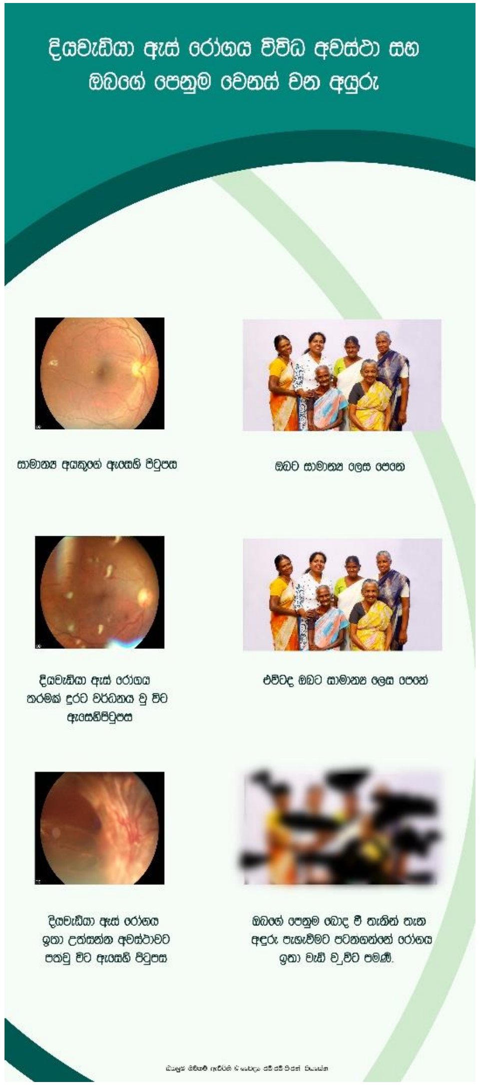

Copy Right Holder of the Leaflets - ©Dr.M.M.P.N.Piyasena-London School of Hygiene and Tropical Medicine (November/2018)

ඇසේ සුද ඉවත්කිරීමේ සැත්කම් හා ඇස් කණ්ණාඩි මගින් දියවැඩියා ඇස් රෝගය වලක්වා ගත හැකිද?

- දියවැඩියා ඇස් රෝගය, ඇසෙහි සුද හෝ කණ්ණාඩි පැළඳීම සමග පටලවා නොගත යුතුය.
- අප කණ්ණාඩි කුට්ටමක් පැළඳවුයේ ඇසෙහි ඉදිරිපසින් ඇති කළු ඉංගිරියාවේ හෝ කාචයේ වෙනස්කම් මග හරවා ගැනීමටයි.
- සුදු ඉවත්කිරීමේ සැත්කම සිදුකරනු ලබන්නේ ඇසෙහි කාචය ඝණකම් වී විනිවිද නොපෙනෙන මට්ටමට පත්වූ විටයි.
- එමනිසා ඇසේ සුද ඉවත්කිරීමේ සැත්කමින් හෝ කණ්ණාඩි මගින් දියවැඩියා ඇස් රෝගය වලක්වා ගත නොහැකිය.
- එසේම බෙහෙත් බිංදු, අත් බෙහෙත්,ආදිය මගින් දියවැඩියා ඇස් රෝග වලක්වා ගත නොහැකිය.
- එමනිසා ඔබ ඇසේ සුද ඉවත්කිරීමේ සැත්කම් සිදුකල පසුවත්, ඇස්කණ්ණාඩි පැළඳිම සිදුකලත්, (වෙනත් ඇස් රෝග ප්‍රතිකාර සිදුකර ඇතත්) දියවැඩියා ඇස් රෝගය පෙර හඳුනා ගැනිමේ පරීක්ෂාවට සහභාගී විය යුතු වේ.

දියවැඩියා ඇස් රෝගය පෙර හඳුනා ගැනීමේ පරීක්ෂණයට මුල් අවස්ථාවේදී ක්‍රමවත්ව සහභාගීවීමේ ඇති වැදගත්කම කුමක්ද?

- දියවැඩියා ඇස් රෝගය ප්‍රධාන ලක්ෂණයක් වන්නේ, මුල් අවස්ථාවලදී කිසිදු රෝග ලක්ෂණයක් නොපෙන්වීමයි.
- පෙර හඳුනාගැනීමේදී ඇස් පරීක්ෂාවක් මගින් ඔබ රෝග ලක්ෂණ දැන ගැනීමට පෙර රෝගය හඳුනා ගතහැකිවේ.
- මෙම වෙනස්කම් කල්තියා හඳුනාගෙන ප්‍රතිකාර කළහොත්, එය සාර්ථක වන අතර එමගින් පෙනුම අඩුවීම හෝ අන්ධභාවය වලක්වා ගත හැකිවේ.
- එමනිසා ක්‍රමානුකූලව ඇස් පරීක්ෂා කිරීම ඉතා වැදගත්වේ.

වෛද්‍ය සායනයේදී ඔබගේ ඇස් පරීක්ෂා කරන්නේ කෙසේද?

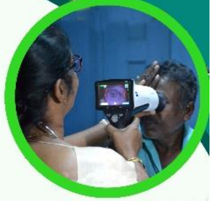

- ඔබගේ වෛද්‍යවරයා විසින් විශේෂිත කැමරාවක් මගින් දියවැඩියා ඇස් රෝගය පරීක්ෂාකරනු ඇත.

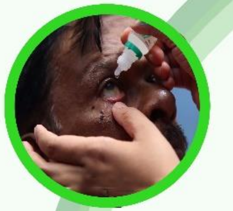

- එහිදී ඇසෙහි ඇතුලත පිටුපස හොඳින් පරීක්ෂා කිරීමට ඇසට බිංදු දමනු ලැබේ.
- ඇසට බිංදු දැමීමෙන් පසුව ඔබට සුළු අපහසුතා ඇතිවුවද, එමබිංදු වල ක්‍රියාකාරීත්වය පැය කිහිපයකින් පහවයන බැවින්, ඔබ ඒ සඳහා බිය විය යුතු නැත.

වෛද්‍ය සායනයේදී ඇස් පරීක්ෂාවෙන් පසුය ඔබ සිදුකළයුත්තේ කුමක්ද?

- දියවැඩියා ඇස් රෝග පරීක්ෂාවේ ප්‍රතිඵල වෛද්‍යවරයා විසින් පවසනු ඇත.
- එම පරීක්ෂාවෙන් පසුව ඔබට දියවැඩියා ඇස් රෝග ලක්ෂණ ඇත්නම් වැඩිදුර පරීක්ෂාවක් සඳහා කොළඹ ජාතික අක්ෂි රෝහල වෙත යොමුකරනු ලැබේ.

එසේ නැතහොත්,

- එහිදී ඔබට දියවැඩියා ඇස් රෝග ලක්ෂණ දැනට නොමැතිනම් හෝ මෙම අවස්ථාවේ ඔබට ඇත්තේ සුළු රෝග ලක්ෂණ පමණක් නම් වසරකින් නැවත ඇස් පරීක්ෂා කළයුතු බව දන්වනු ලැබේ.
- මෙහිදී දියවැඩියා ඇස් රෝගය සඳහා පමණක් පරීක්ෂා කරන බැවින් වෙනත් ඇස් රෝග ඇත්නම්, ඒ සඳහා අදාල සායනය වලට සහභාගී වියයුතු බව සලකන්න.

Copy Right Holder of the Leaflets - ©Dr.M.M.P.N.Piyasena-London School of Hygiene and Tropical Medicine (November/2018)

අක්ෂි සායනය වෙත යාමට අනුගමනය කළයුතු පියවර:

- කොළඹ ජාතික අක්ෂි රෝහලේ මෙම දියවැඩියා ඇස් රෝගය සඳහා අවශ්‍ය නවීනතම පරීක්ෂණ හා ප්‍රතිකාර ක්‍රම ඇත.

- දියවැඩියා ඇස් රෝගය සඳහා විශේෂඥ වෛද්‍යවරු විසින් එහිදී ඔබට ප්‍රතිකාර කරනු ඇත.

අක්ෂි සායනයට යාම සඳහා පහත මාර්ග සටහන අනුගමනය කරන්න.

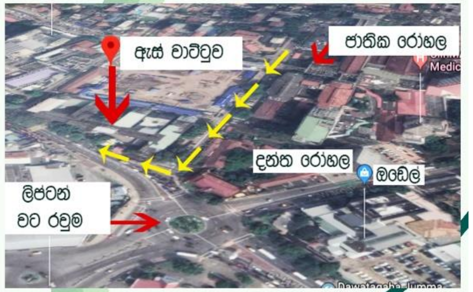

අක්ෂි සායනය ඔබ සිදු කළයුත්තේ කුමක්ද?

ගේට්ටු අංක 4න් ඇස් රෝහලට ඇතුළු වී OPD/ඕපීඩී අංශ නිකුත් කරන කවුළුව වෙත යන්න.

එහිදී ඔබගේ නම,ගම වයස විස්තර ලබා දී බාහිර රෝගී අංශයේ සායනික තුණ්ඩුවක් ලබා ගන්න.

ඉන්පසු මූලික පෙනුම පරීක්ෂා කරන කාමර අංක(10) වෙත යන්න.

පෙනීම පරීක්ෂා කළ පසුව ඔබගේ අංකයේ අනුපිළිවෙල අනුව අදාල අක්ෂි සායනය වෙත යොමුකරනු ඇත.

- ඉන්පසුව අක්ෂි වෛද්‍යවරයා විසින් ඉදිරියට ගත යුතු පියවර හා ප්‍රතිකාර පිළිබඳව ඔබට උපදෙස් ලබාදෙනු ඇත.

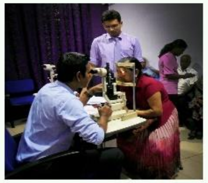

ඔබ නැවත ඇස් පරීක්ෂාවට හෝ වැඩිදුර පරීක්ෂා/ප්‍රතිකාරවලට පැමිණෙන විට මතක තබා ගත යුතු කරුණු

- ඔබ කණ්ණාඩි පාවිච්චි කරන්නේ නම් කණ්නාඩි කුට්ටම හෝ සිවි කාච පාවිච්චි කරන්නේ නම් සිවි කාච සහ අදාල දියරය රැගෙන එමට මතක තබාගන්න.

- තවත් අයකු සමග පැමිණෙන ලෙස උපදෙස් දුන් විට ඒ අනුව ක්‍රියාකරන්න.

- ඇසට දියර බිංදු දැමීමෙන් පසු පැය කිහිපයක් යනතුරු සුළු බොඳ වීමක් ඇතිවන බැවින් රිය පැදවීම/ බයිසිකල්/ත්‍රී රෝද පැදවීම සිදුනොකළ යුතුයි.

- රෝහලෙන් පිටවීමට පෙර ඉදිරි පියවර පිළිබඳව අක්ෂි වෛද්‍යවරයාගෙන් පැහැදිලිව අසා දැනගත යුතුයි.

- මෙහිදී නැවත පැමිණිය යුතු දිනය, වේලාව,කාමර අංකය,පිටුපස පිටුවෙහි සටහන් කරගන්න.

- ඊළඟට සායනයට පැමිණෙන විට ඔබගේ සායන තුණ්ඩුවේ අංකය,පසුගිය ඇස් පරීක්ෂණ වාර්තා ගෙන එමට මතක තබා ගන්න.

දිනය	රෝහලේ නම	සායන කාමර අංකය

LONDON SCHOOL of HYGIENE & TROPICAL MEDICINE

Copy Right Holder of the Leaflets - ©Dr.M.M.P.N.Piyasena-London School of Hygiene and Tropical Medicine (November/2018)
