# Supplementary material for: Process of adaptation, development and assessment of acceptability of a health educational intervention to improve referral uptake by people with diabetes in Sri Lanka
Source: BMC Public Health. 2019 May 21;19:614. doi: 10.1186/s12889-019-6880-4 (PMC6528317; doi:10.1186/s12889-019-6880-4)
Supplement: Supplementary file 2 — 2.1: Leaflet health educational intervention in English and local languages (Sinhala andTamil) and 2.2: Outline and script in English and local languages (Sinhala and Tamil) of the video healtheducational Intervention. (ZIP 3663 kb) [file 12889_2019_6880_MOESM2_ESM.zip › 5_BMC_HEI_Additional File 2.2_Video_R2.pdf]

Additional File 2 - Outline and script in English and local languages (Sinhala and Tamil) of the video health educational Intervention

<table>
<tr><th>Video Picture Frames</th><th>English</th><th>Sinhala</th><th>Tamil</th></tr>
<tr><td>1st Segment</td><td></td><td></td><td></td></tr>
<tr><td>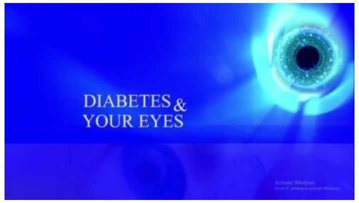
</td><td>Run time in seconds - 00:01
Diabetes and your eyes.</td><td>දියවැඩියාවයි ඔබේ ඇසයි.</td><td>'நீரிழிவும் உங்கள் கண்களும்'</td></tr>
<tr><td>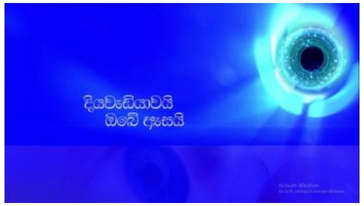
</td><td></td><td></td><td></td></tr>
<tr><td>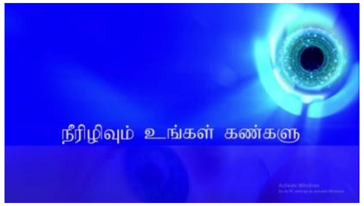</td><td>00:04
Sugar disease harms your eye sight.</td><td>ඇසේ පෙනුමට හානිකර සීනි.</td><td>“பார்வையை பறிக்கும் சீனி“</td></tr>
<tr><td>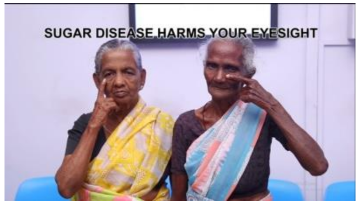
</td><td>00:06
Your guide to protect your eye sight from diabetic retinopathy or diabetic eye ailment.
00:15</td><td>දියවැඩියා ඇස් රෝගයෙන් පෙනුම අඩුවීම හා අන්ධවීම වලක්වා ගැනීමට ඔබට උපදෙස්.</td><td>நீரிழிவுக்கான கண் நோயிலிருந்து உங்கள் கண்களை பாதுகாப்பதற்கான வழிகாட்டி</td></tr>
</table>

Copy Right Holder of the Video © Dr.M.M.P.N.Piyasena – London School of Hygiene and Tropical Medicine – UK

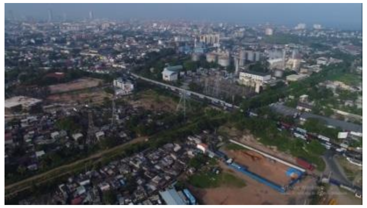<br>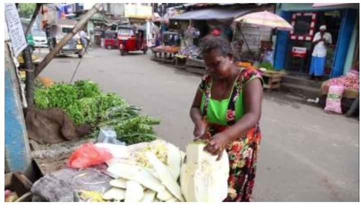<br>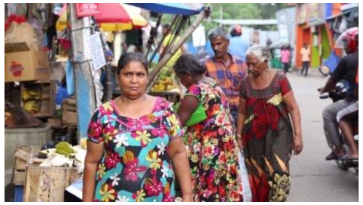	**Are you aware that diabetes causes visual loss?**<br><br>00.19<br><br>In Sri Lanka for every 100 people above 20 years of age at least 20 people have diabetes.<br><br>00.28<br><br>That means in an average family of 5 people 1 could have diabetes.<br><br>Substantial number of these people's eyes could've already been affected by diabetes.<br><br>For every 3 people with diabetes, 1 can have diabetic eye ailment. You could be one of them.	**දියවැඩියාවෙන් ඇස් පෙනීම අඩුවීමට හෝ අන්ධවීමට හැකිබව ඔබ දන්නවාද?**<br><br>ශ්‍රී ලංකාවේ වයස අවුරුදු 20ට වැඩි සියදෙනෙකුගෙන් විසි දෙනෙකුට පමණ දියවැඩියාව ඇත.<br><br>ශ්‍රී ලංකාවේ එක පවුලක වැඩිහිටි පස් දෙනෙකු සැලකූ විට ඉන් එක් අයෙක් දියවැඩියා රෝගියෙකු විය හැක.<br><br>ඔවුන්ගෙන් වැඩි පිරිසකගේ ඇස් දියවැඩියාවෙන් හානි වී අන්ධභාවයට පත්වීමේ හැකියාව ඇත.<br><br>ඔවුන්ගෙන් සෑම තුන්දෙනෙකුගෙන් එක් අයෙකුම දියවැඩියා ඇස් රෝගයෙන් පෙලෙන අතර ඔබ එයින් එක් අයෙකු විය හැකිය.	நீரிழிவினால் கண்பார்வையை இழக்க நேரிடலாம் என்பது உங்களுக்கு தெரியுமா?<br><br>இலங்கையில் 20 வயதுக்கு மேற்பட்ட ஒவ்வொரு நூறு பேரில் ஆகக்குறைந்தது இருபது பேர் நீரிழிவினால் பாதிக்கப்பட்டிருக்கின்றனர்.<br><br>இலங்கையில் ஒரு குடும்பத்தில் வளர்ந்தோர் 5 பேரில் ஒருவர் நீரிழிவு நோயாளராக இருக்கலாம்<br><br>இவர்களின் குறிப்பிடத்தக்களவு நபர்களின் கண்கள் ஏற்கனவே நீரிழிவினால் பாதிக்கப்பட்டிருக்கலாம்.<br><br>அவர்களில் மூவரில் ஒருவருக்கு நீரிழிவுக் கண்

Copy Right Holder of the Video © Dr.M.M.P.N.Piyasena – London School of Hygiene and Tropical Medicine – UK

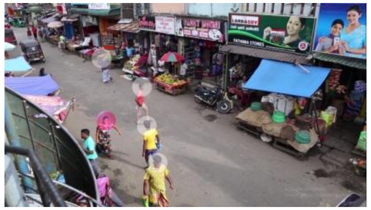			நோய் இருக்கலாம் என்பதோடு நீங்கள் அதில் ஒருவராக இருக்கலாம்.
**2nd Segment**<br>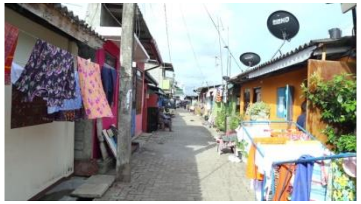<br>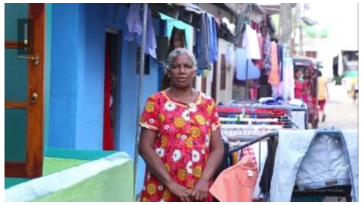	00:50<br>**What is diabetic eye ailment?**<br>00:54<br>*I have diabetes for more than 20 years, one day I lost vision in my right eye suddenly. And I presented to emergency eye clinic at eye hospital.*<br>*There doctors found a sudden bleeding inside my eye due to diabetes. I underwent a big operation to clear the blood inside my eye.*	**දියවැඩියා ඇස් රෝගය යනු කුමක්ද?**<br>මට දියවැඩියාව හැදිලා දැන් අවුරුදු 20ක් වෙනවා. හිටපු ගමන් මගෙ දකුණු ඇහේ පෙනීම නැතිවෙලා ගියා.<br>මම එවෙලෙම ඇස් රෝහලේ හදිසි ප්‍රතිකාර ඒකකයට ගියා.<br>දොස්තරලා මට කිව්වේ දියවැඩියාව නිසා ඇසේ ඇතුලට ලේ ගලලා පෙනීම නැතිවෙලා කියලා. ඊලඟට මගේ ඇහේ ලොකු සැත්කමක් කරලා ලේ ටික අයින් කලා.	நீரிழிவுக் கண் நோய் என்றால் என்ன?<br>எனக்கு நீரிழிவுக் கண்நோய் ஏற்பட்டு 20 வருடங்களுக்கு மேலாகின்றது. ஒருநாள் திடீரென்று எனது வலது கண் தெரியாமல் போனது. உடனே கண் வைத்தியசாலையில் அவசர சிகிச்சை பிரிவுக்கு சென்றேன்.<br>நீரிழிவினால் கண்ணின் உட்புறத்தில் இரத்தம் கசிந்து கண் தெரியாமல் போய்விட்டது என்று வைத்தியர்கள் என்னிடம்

Copy Right Holder of the Video © Dr.M.M.P.N.Piyasena – London School of Hygiene and Tropical Medicine – UK

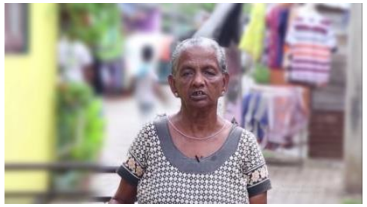	*Last 20 years I was always concentrating on my family matters and I could not pay much attention to my own health. There I got to know that this type of an incident could have been prevented at an early stage.*	පහුගිය අවුරුදු 20, 30 ඇතුලත ගොඩක් පවුලේ ප්‍රශ්ණ තිබුන නිසා මගේ ලෙඩ ගැන මට බලන්න බැරිව ගියා. එදා තමයි මම දැනගත්තේ අවුරුද්දක් පාසා ඇස් බලන්න ඕන කියලා.	கூறினார்கள். கண்ணில் பெரிய சத்திர சிகிச்சை செய்து இரத்தத்தை அகற்றினார்கள்.<br><br>கடந்த 20 வருடங்களாக நான் எனது குடும்ப பிரச்சினை பற்றி மாத்திரமே கவனம் செலுத்தியதனால் என்னை பற்றி கவனத்தில் எடுக்கவில்லை.<br><br>வருடா வருடம் கண்ணை பரிசோதித்துக்கொள்ள வேண்டும் என்பதனை அன்று தான் நான் தெரிஞ்சுகொண்டேன்.
**3rd Segment**<br>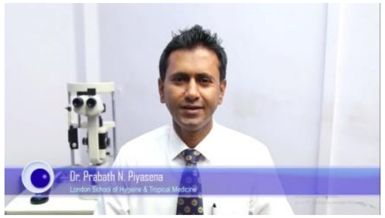 	01.05<br><br>*High blood sugar due to diabetes lead to diabetic eye disease. Diabetes affects the blood tubes which supply the light sensitive layer at the back of the eye which is called retina.*	දියවැඩියාව නිසා ලේ වල ඇති අධික සීනි මට්ටම දියවැඩියා ඇස් රෝගයට හේතු වෙනවා. එමඟින් ඔබේ ඇසට පෙනුම ලබාදෙන ඇසේ පිටුපස ඇති ස්නායු පටලය එහෙම නැත්නම් දෘෂ්ටි විතානයේ ලේ නහරවලට බලපෑම් ඇතිකරනවා.	"இரத்தத்தில் அதிகளவான சீனி இந்த நீரிழிவுக் கண் நோய் ஏற்பட வழிவகுக்கின்றது நீரிழிவு நோயானது கண்ணின் பின்புறத்திலுள்ள பார்வையை வழங்கும் நரம்பு மண்டலத்திலுள்ள

Copy Right Holder of the Video © Dr.M.M.P.N.Piyasena – London School of Hygiene and Tropical Medicine – UK

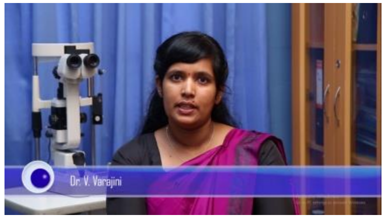<br><br>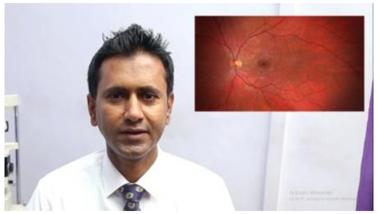<br>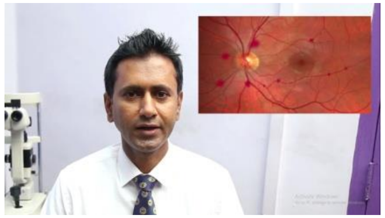<br>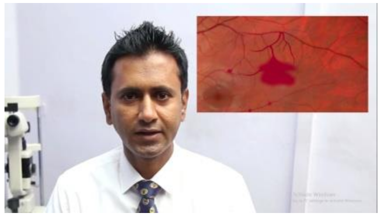	01.29<br><br>*These blood tubes start bleeding in diabetes. Then weak blood tubes grow abnormally at the back of your eyes then bleed or leak which you cannot see from outside affecting your eye sight.*<br><br>*This can progress in to vision loss and blindness if not detected and treated at correct stage.*	පළමුව මේ ලේ නහර වලින් ඉතා සුළු වශයෙන් රුධිර වහනය වීමට පටන්ගන්නවා. ඉන්පසුව මෙම රුධිර නාල දුර්වල වී අසාමාන්‍ය ලෙස වර්ධනය වී තදුරටත් රුධිර ගැලීම් හෝ රුධිර කාන්දු වීම් සිදුවී, ඔබගේ පෙනීමට හානි සිදුවෙනවා.<br>මෙය ඇසේ පිටුපසින් සිදුවන නිසා ඔබට ඇසේ ඉදිරිපසින් මෙය බලාගත නොහැකි.<br><br>මෙය හරි වේලාවට හඳුනාගෙන ප්‍රතිකාර කලේ නැත්නම් ඔබගේ පෙනුම අඩුවීමට හෝ අවසානයේ අන්ධභාවයට වුනත් පත්වෙන්න පුළුවන්.	இரத்தக் குழாய்களை பாதிக்கின்றது.<br><br>நீரிழிவு நோயின்போது இந்த இரத்தக் குழாய்களிலிருந்து இரத்தம் கசிய ஆரம்பிக்கும். பின்னர் இந்த பலவீனமான இரத்தக் குழாய்கள் அசாதாரணமான முறையில் வளர்ச்சியடைந்து இரத்தம் சிந்துவதனால் அல்லது கசிவதனால் கண்பார்வை பாதிப்படைவதுடன் அதனை வெளிப்புறத்திலிருந்து காண முடியாது.<br><br>சரியான நேரத்தில் அடையாளம் கண்டு சிகிச்சை பெறாவிட்டால் இந்நிலை மோசமடைந்து கண்பார்வை குறைவடைவதற்கு அல்லது இழப்பதற்கு அது வழிவகுக்கலாம்.“.

Copy Right Holder of the Video © Dr.M.M.P.N.Piyasena – London School of Hygiene and Tropical Medicine – UK

**4th Segment**	02.07<br><br>**How would your eyes be checked at the medical clinic?**	**වෛද්‍ය සායනයේදී ඔබගේ ඇස් පරික්ෂාකරන්නේ කෙසේද?**	மருத்துவ பரிசோதனையில் உங்கள் கண்கள் எவ்வாறு பரிசோதிக்கப்படும்?
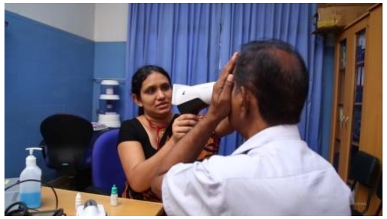<br>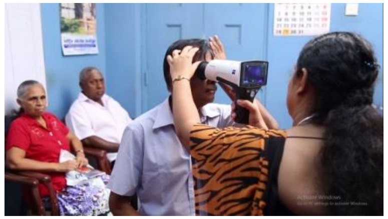<br>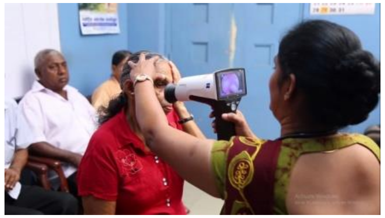	02:11<br><br>Your doctor will examine your eyes using a special eye camera and eye drop will be instilled in your eyes to have a better view of the back of the inside of your eyes. You will have blurring for short period of time. But the outcome of examination is more beneficial than the difficulties. Further, this is a routine method done on everybody that you do not need to worry about.	ඔබගේ වෛද්‍යවරයා විසින් විශේෂිත කැමරාවක් මඟින් ඇස් පරික්ෂා කරනු ඇත. එහිදී ඔබගේ ඇසේ අතුලත පිටුපස හොඳින් පරීක්ෂා කිරීමට ඇසට බිංදු දමනු ලැබේ. එවිට ඇසේ බොඳවීමක් තාවකාලිකව ඇතිවිය හැකිවුවද එම පරික්ෂණයේ ප්‍රථිපල එම ඇතිවන අපහසුතාවට වඩා වාසි සහගත වේ. තවද මෙය සෑම අයෙකුගේම නිතර සිදුකරන පරික්ෂාවක් බැවින් ඔබ ඒ සඳහා බියවිය යුතු නැත.	“உங்கள் வைத்தியர் விசேட கமரா ஒன்றின் மூலம் நீரிழிவுக் கண் நோய் இருக்கின்றதா என பரிசோதித்துப் பார்ப்பார்.<br><br>கண்ணின் பின்பகுதியினை உட்பக்கத்தால் தெளிவாக பார்ப்பதற்கு விசேட சொட்டு மருந்து சில துளிகள் கண்ணுக்கு விடப்படும். பார்வை தற்காலிகமாக மங்களாக தெரியும் என்றாலும் அந்த அசௌகரியத்தை விட பரிசோதனையின் பலன் அதிகமானதாகும். மேலும் இது எல்லோருக்கும் வழமையாக மேற்கொள்ளும் பரிசோதனை என்பதனால்

Copy Right Holder of the Video © Dr.M.M.P.N.Piyasena – London School of Hygiene and Tropical Medicine – UK

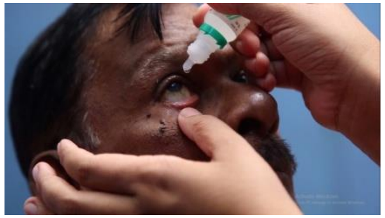<br>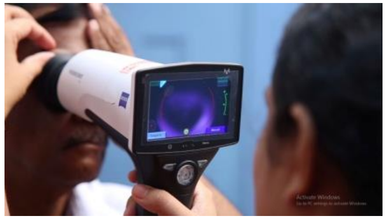			அது தொடர்பாக பயப்படத் தேவையில்லை“
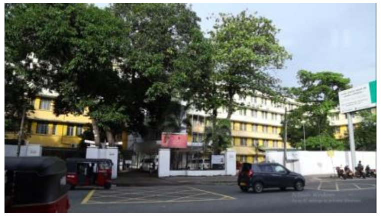	02:38<br>**What you will have to do next?**<br>02:43<br>Small number of people will be referred to eye clinic of the National Eye Hospital Colombo, if found to have the disease or to have a better look at your eyes. If not, you will be asked to go under go same eye examination at the medical clinic in one year time.	**වෛද්‍යසායනයේ ඇස් පරික්ෂාවෙන් පසුව ඔබ සිදුකල යුත්තේ කුමක්ද?**<br>දියවැඩියා ඇස් රෝගය ඇති සුළු පිරිසක් තවදුරටත් ඇස් ඇතුලත හොඳින් පරික්ෂා කිරීමට කොළඹ ජාතික අක්ෂි රෝහලේ ඇස් සායනයට යොමුකරනු ඇත. එසේ නැතහොත්, ඔබට දියවැඩියා ඇස් රෝග ලක්ෂන දැනට නොමැතිනම් හෝ මෙම අවස්ථාවේ ඇත්තෙ සුළු රෝග ලක්ෂණ පමනක් නම් නැවත වසරකට පසු පරික්ෂා කලයුතුබව පවසනු ඇත.	நீங்கள் அடுத்து என்ன செய்வீர்கள்?<br>நீரிழிவுக் கண் நோய் அறிகுறிகள் இருந்தால் அல்லது கண்களை மேலும் சிறந்த முறையில் பரிசோதித்துப் பார்ப்பதற்கு சிறு அளவிலான நபர்களை தேசிய கண் வைத்தியசாலையின்

Copy Right Holder of the Video © Dr.M.M.P.N.Piyasena – London School of Hygiene and Tropical Medicine – UK

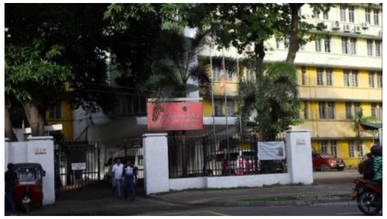			மருத்துவ பரிசோதனைக்கு சிபாரிசு செய்து அனுப்பப்படுவீர்கள்.<br><br>அவ்வாறு இல்லாவிட்டால் இந்த கண் பரிசோதனையை ஒரு வருடத்தின் பின்னர் மீண்டும் செய்துகொள்ளுமாறு கேட்டுக்கொள்ளப்படுவீர்கள்.
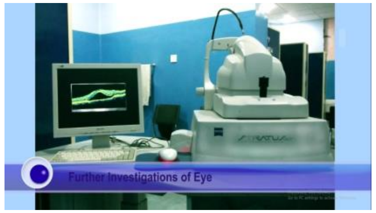	03:01<br><br>**Why should you undergo eye examination at National Eye Hospital?**<br><br>03:06<br><br>National Eye Hospital Colombo has all the advanced investigation and treatment facilities for this condition. There you will be well instructed on what to do next depending on the status of your eyes or found to have advanced eye problem.	**ඔබ කොළඹ ජාතික අක්ෂි රෝහලේ ඇස් පඉක්ෂාවට සහභාගී විය යුත්තේ ඇයි?**<br><br>කොළඹ ජාතික අක්ෂි රෝහලේ මෙම දියවැඩියා ඇස් රෝගය සඳහා අවශ්‍ය නවීනතම පරීක්ෂන හා ප්‍රතිකාර ක්‍රම ඇත. එහිදී, ඔබගේ රෝගයේ තත්වය අනුව හෝ උත්සන්නභාවය අනුව මීලඟට සිදුකලයුතු දෑ පිලිබඳව උපදෙස් ලබාදෙනු ඇත.	தேசிய கண் வைத்தியசாலையில் கண் மருத்துவ பரிசோதனையினை ஏன் செய்துகொள்ள வேண்டும்?<br><br>கொழும்பு தேசிய கண் வைத்தியசாலையில் நீரிழிவு கண் நோய்க்கான சகலவித முன்னேற்றகரமான பரிசோதனைகளுக்கும் சிகிச்சைகளுக்குமான

Copy Right Holder of the Video © Dr.M.M.P.N.Piyasena – London School of Hygiene and Tropical Medicine – UK

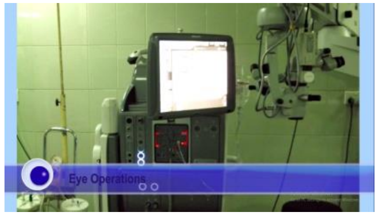			வசதிகள் உள்ளன. அங்கு, உங்களின் நீரிழிவுக் கண்ணோயின் தீவிரத் தன்மைக்கேற்ப அடுத்த என்ன செய்ய வேண்டும் என்பது தொடர்பாக சரியான முறையில் வழிநடாத்துவார்கள்.
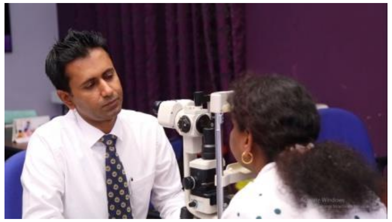<br>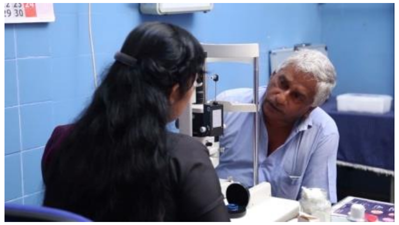	*03:24*<br>*You have diabetic eye disease which require treatment. We have to start treatments immediately.*<br>*03:30*<br>*Doctor, I have undergone cataract surgery and I am using spectacles as well. I do not have any problem with my sight.*<br>03:40	ඔබට දියවැඩියා ඇස් රෝගය ප්‍රපීකාර කලයුතු මට්ටමට තියනවා ඉක්මිනින්ම ප්‍රථ්කාරවලට යොමුවෙන්න ඕන.<br>ඩොක්ටර්, මගෙ ඇස්වල සුද හැදිලා ඔපරේෂන් කරලා කාච දාල තියෙන්නේ, මම කන්නාඩිත් පාවිච්චි කරනවා. මගේ ඇස්වල පෙනීම කිසිම ප්‍රශ්ණයක් නෑනේ.	உங்களுக்க சிகிச்சை பெற வேண்டிய அளவில் நீரிழிவுக் கண் நோய் இருக்கின்றது. உடனடியாக சிகிச்சையை ஆரம்பிக்க வேண்டும்.<br>டாக்டர், கண்ணில் வெண்படல அறுவை சிகிச்சை செய்து கண்ணாடியும் பாவிக்கின்றேன் தானே. பார்வையில் எந்தவித பிரச்சினையும் இல்லையே.

Copy Right Holder of the Video © Dr.M.M.P.N.Piyasena – London School of Hygiene and Tropical Medicine – UK

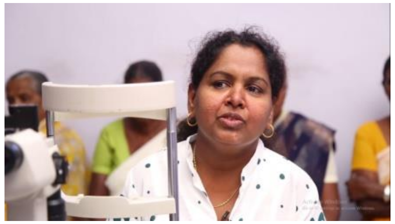	*This can occur with another eye disease so treating one condition does not protect against diabetic eye disease.*<br><br>03:49	දියවැඩියා ඇස් රෝගය වෙනත් රෝග සමඟ ඇතිවිය හැකි අතර එම රෝගවලට කරන ප්‍රතිකාරවලින් දියවැඩියා ඇස් රෝගය සුවවන්නේ නෑ.	நீரிழிவுக் கண் நோயானது வேறு கண் நோய்களுடனும் ஏற்படலாம். எனினும் அவற்றுக்கு எடுத்துக்கொள்ளும் சிகிச்சை மூலம் நீரிழிவுக் கண் நோயை குணப்படுத்த முடியாது<br>.
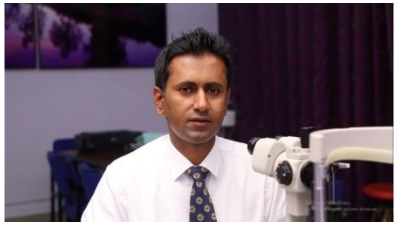	**Why is it important to check early and regularly for diabetic eye ailment?**<br><br>03:56	**දියවැඩියා ඇස් රෝගය පෙරහඳුනා ගැනිමේ පරීක්ෂනයට මුල් අවස්ථාවෙම හා ක්‍රමවත්ව සහභාගි වීමේ වැදගත්කම කුමක්ද?**	நீரிழிவுக் கண் நோய் கண்டுபிடிக்கும் பரிசோதனையை ஆரம்பகட்டத்திலும் ஒழுங்கு முறையாகவும் செய்துகொள்வது ஏன் முக்கியமானது?
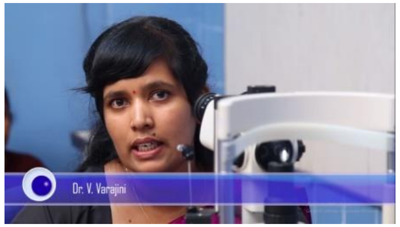	*Main feature of this condition is, you would not feel any symptoms related with diabetic eye disease at early stages. Testing early or screening can detect changes at back of your eyes at an early stage before you aware of them. Treatments can work very well, if detected and treated.*	ඔබ දැනගත යුතුයි දියවැඩියා ඇස් රෝගයේ ප්‍රදානතම කරුනක් වන්නේ මුල් අවස්ථාවලදී කිසිඳු රෝග ලක්ෂණයක් නොපෙන්වීමයි. පෙර හඳුනාගැනීමේ පරික්ෂාවක් මඟින් ඔබ රෝග ලක්ෂ්ණ දැනගැනීමට පෙර රෝගය හඳුනාගත හැකි වෙනවා. මුල් අවස්ථාවේම රෝගය හඳුනාගෙන ප්‍රතිකාර කිරීම මඟින් එම ප්‍රතිකාර ඉතා සාර්තකවන අතර අන්ධභාවය වලක්වාගන්න පුළුවන්.	இந்த நோயின் பிரதான பண்பு அதன் ஆரம்ப கட்டங்களில் அது தொடர்பான எந்தவொரு அறிகுறியையும் காட்டாதிருப்பதாகும். நீங்கள் அந்த நோய் அறிகுறிகளை உணர்வதற்கு முன்னரே அதற்கான

Copy Right Holder of the Video © Dr.M.M.P.N.Piyasena – London School of Hygiene and Tropical Medicine – UK

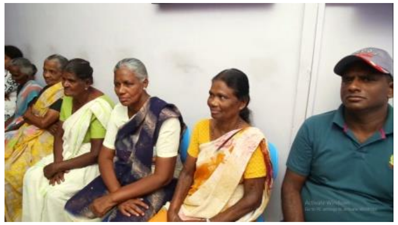 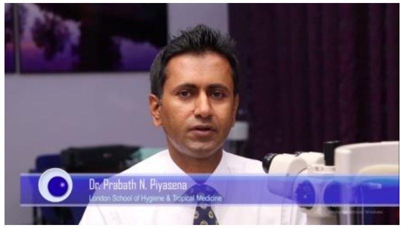			கண்டுபிடிப்பு பரிசோதனையின் மூலம் அதனை அடையாளம் காணலாம். இம்மாற்றங்களை முன்கூட்டியே அடையாளம் கண்டு சிகிச்சை பெற்றால் அது வெற்றியளிக்கலாம்.
	04:18<br>*Now I know this could have been prevented by early eye checking and treatment. I know one of my friends lost vision in both eyes. He had undergone lens implantation as well. However, now he has lost vision in both eyes due to this diabetic eye ailment and he has very*	එහෙම කරලා ප්‍රතිකාරයක් කෙරුවානම් මෙහෙම දෙයක් වෙන්නේ නෑ. මගේ යාළුවෙක් ඉන්නවා ඇස් දෙකම පේන්නේ නෑ. එයාව ඇස් වාට්ටු ගෙනිල්ලා කාව දැම්මා. දැන් දියවැඩියාව නිසා ඇස් දෙකම නරක් වෙලා. ඒක නිසා පෙනීම දුර්වලම වෙලා. මම ඔයාලා	கண்களை ஆரம்ப கட்டத்தில் பரிசோதனை செய்து அதற்கு சிகிச்சை எடுத்திருந்தால் இதனை தடுத்துக்கொள்ள இருந்தது

Copy Right Holder of the Video © Dr.M.M.P.N.Piyasena – London School of Hygiene and Tropical Medicine – UK

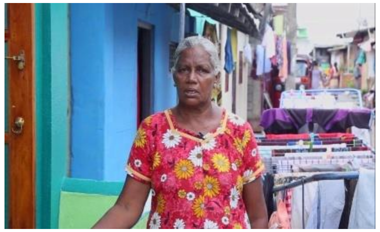<br>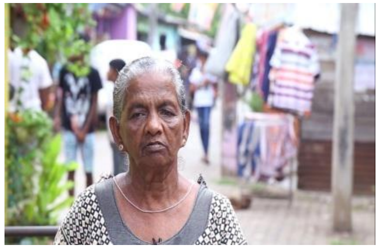<br>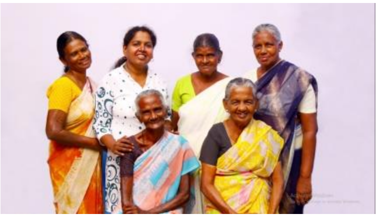	*weak eyes. Therefore, I urge all of you to go annual eye checking as we celebrate Sri Lankan New Year in each year in April. Protect your eyes from this diabetic eye ailment.*<br><br>04:55 (End)	සියලු දෙනාටම කියන්නේ අවුරුද්දෙන් අවුරුද්ද අප්‍රේල් මාසේ සිංහල දෙමළ අලුත් අවුරුද්ද සමරනවා වගේ අවුරුද්දක් පාසා ඇස් රෝහලට ගිහින් ඇස් පරීක්ෂා කරලා මේ දියවැඩියා රෝගයෙන් ඇස් දෙක පරිස්සම් කරගන්න කියලා.	என்று இப்போது எனக்கு தெரியும்.<br><br>எனக்கு தெரிந்த ஒரு நண்பர் இருக்கின்றார் அவரது இரண்டு கண்களது பார்வையும் இழந்துவிட்டார். அவர் கண்களுக்கு வில்லையும் பொருத்தி இருக்கிறார். என்றாலும் நீரிழிவு கண் நோய் காரணமாக இரண்டு கண்களும் தெரியாமல் போயுள்ளது. கண்கள் இப்போது மிகவும் மோசமான நிலையில் உள்ளது. எனவே வருடா வருடம் சித்திரை மாதம் சிங்கள தமிழ் புத்தாண்டை கொண்டாடுவது போல வருடா வருடம் கண்களையும் பரிசோதித்து இந்த நீரிழிவு நோயிலிருந்து கண்களை பாதுகாத்துக்கொள்ளுமாறு வேண்டிக் கொள்கிறேன்.

Copy Right Holder of the Video © Dr.M.M.P.N.Piyasena – London School of Hygiene and Tropical Medicine – UK
