# Supplementary material for: Process of adaptation, development and assessment of acceptability of a health educational intervention to improve referral uptake by people with diabetes in Sri Lanka
Source: BMC Public Health. 2019 May 21;19:614. doi: 10.1186/s12889-019-6880-4 (PMC6528317; doi:10.1186/s12889-019-6880-4)
Supplement: Supplementary file 3 — Topic guides for field testing of the HEI – for service users and service providers. (DOCX 18 kb) [file 12889_2019_6880_MOESM3_ESM.docx]

**Additional File 3 – Topic guides for field testing of the HEI – for service users and service providers**

[Version 1.2_June/2018]

Date and Time of the SSI – …………………………………………

Names of the investigators –……………………………………….

Place of the interview – …………………………………………….

Participant study ID-………………………………………………...

**Introduction –**

*Background information –*

*“Ayubowan (Greeting), …Introduction of the moderator and the investigator…”*

*“Do you remember you received a leaflet and video 2-3 weeks ago when you were in the medical clinic. Today we want to get your opinion on these, because, as you know, this sugar/diabetic eye ailment is a major problem in our country. It is important to us to understand what people think about these materials and how we might improve them.*

*Now you know that diabetes can affect your eyes, as it affects your kidneys. The high sugar levels in your body, due to sugar disease, leads to changes at the back of your eyes leading to vision loss. However, this can be prevented if you checked your eyes on time.*

*The things we discuss will be very confidential and we will not be attaching your name or any details that anyone can identify you when reporting your answers. You can also stop the interview at any time if you wish”.*

*Instructions - The leaflet and video should be assessed separately 1^st^ – Leaflet, 2^nd^ Video

**Additional file 3 - Table 1. Topic guide for PwDM**

| **General Topic Guide** | **Specific topic related to Leaflet** | **Specific topic related to Video** |
| --- | --- | --- |
| [Introductory topic]  1.What are the usual sources of getting information on your eye problems? | N/A | N/A |
| [Acceptability]  2.In general, what did you think about the (intervention)? | In general, what did you think about the leaflet?  What did you prefer most (Leaflet/video)?  Do you think you need both? | In general, what did you think about the video?  What did you prefer most (Leaflet / Video)?  Do you think you need both? |
| [Comprehensibility]  3.What did you understand after going through the video / leaflet given to you by your physician few days / few weeks ago | What was the subject explained in the leaflet? | What was the story shown in the video? What was explained by the lady in the video? |
| [Delivery] [Acceptability]  4. How was it delivered to you? | What setting?  Did they take their time to discuss it?  Was there enough time? Did you need extra explanation and time to discuss it?  Probe – How do you feel to receive it at your medical clinic – by your physician?  What is the best place to distribute this leaflet (in a hospital)? Who do you think as the best person to deliver this? Where? And What setting? Why do you think like that?  Probe – What are your thoughts on place of delivery and person who delivered the HE intervention? | What setting?  Did they take their time to discuss it?  How the video was shown to you?  Probe – How do you feel to receive it at your medical clinic – by your physician?  Was there enough time? Did you need extra explanation and time to discuss it / watch it?  What is the best place to show the video (in a hospital)? Where? And What setting? Why do you think like that?  Probe – What are your thoughts on place of delivery and person who delivered the HE intervention? |
|  | *Hand over a leaflet to the participant* | *Start showing the video, segment by segment* |
| [Comprehension and Understandability]  5.What do you understand in each medium (Leaflet and Video separately) in each section? | *[Go through the leaflet page by page – specifically asking about the pictures / diagrams / texts]*  Show me anything that you could not understand?  Probe - Please show me (encircle) the information that you cannot understand properly – in the leaflet?  Any technical or medical terms you found difficult?  What do you understand by the picture in the front-page? Images at 2^nd^ page?  What do you understand the numbers and figures given in the front page?  Can you follow the map given here?  Can you tell me the steps mentioned here in the flow chart – things to be done when you are at eye clinic?  *If not understood / could not follow?*  Why couldn’t you understand those statements / pictures / illustrations? | *[Go through each segment of the video while pausing it at each segment]*  Tell me any segments / statements that could not understand in the video?  Probe - Show me the statements that you could not follow in the video?  Any technical or medical terms you found difficult?  Were you able to understand the animations / graphics shown in describing the developing the diabetic eye ailment? Eye examination at medical clinic? Referral uptake at next level of National Eye Hospital?  *If not understood / could not follow?*  Why couldn’t you understand those statements / animations / video segments? |
| [Readability] | Overall did you find it easy to read?  Probe - Language style, usage of terms, font size? Were you able to read the text easily? Were the size of the letters large enough for you?  Probe – What do you think about the layout and design of the leaflet  For illiterate people – How did you manage to readout it for you? Who supported you in that? What were the difficulties you had in that? | Was it easy to follow the dialogues and statements said in the video?  Probe – What do you think about the narration in the video? Do you like the story?  Any technical terms you found difficult? |
| [Actionability]  6.What is the key message that you got in each intervention in seeking care at eye clinic? | What do you think are the key messages that you will take away from this?  What is the message about referrals? Is it clear? | What do you think are the key messages that you will take away from this?  What is the message about referrals? Is it clear? |
| [Usability]  7. Did you use the given material at home or anywhere else (shared with friends / neighbours / other patients at clinic)? | Did you take the leaflet home and share it with the family members?  Did anyone read it? What did they say?  If so, how did that go? Any difficulties?  If not – Why? | Did you take the given DVD?  Do you have facilities to watch it?  What were difficulties you had in viewing the video?  If not shared – Why? |
| [Suggestions]  8.Any suggestions to improve? | Would you like to suggest any modifications to the leaflet to make more appealing to our community / culture?  Probe – Your suggestions to gain confidence in the diabetic community in order to use this health educational intervention.  Probe – Does it need to be different for men / women; older men / women ? | Would you like to suggest any modifications to the leaflet to make more appealing to our community / culture?  Probe – Your suggestions to gain confidence in the diabetic community in order to use this health educational intervention.  Probe – Does it need to be different for men / women; older men / women ? |
| 9. Anything else important that we haven’t covered in this intervention? | Anything else important that we haven’t covered which you think is important when trying to promote improved uptake of DR assessment at eye surgeons’ clinic following referral?  Overall how you would rate the Leaflet?  Probe – 0 = not very good, 9=excellent | Anything else important that we haven’t covered which you think is important when trying to promote improved uptake of DR assessment at eye surgeons’ clinic following referral?  Overall how you would rate the Video?  Probe – 0 = not very good, 9=excellent |

**Additional file 3 - Table 2. Topic guide for the providers**

| **General topic guide** |
| --- |
| [Introductory topic] |
| 1) How would you describe overall experience of this new health educational intervention?  Probe – Whether you think this would be useful to improve uptake of DR assessment and treatment services at next level of ophthalmologist’s / retinologist’s clinic by the people with referable level DR. |
| [Acceptability] [by the provider – not the patients] |
| 2) What is your opinion on suitability of health educational intervention to medical clinics of the Western province of Sri Lanka?  Probe – Your opinion on suitability of medium of delivery to the context.  Probe – Do you think the medical clinic as the best place to deliver? |
| [Comprehensibility] |
| 3) What is your understanding about appropriateness of content to the context?  Probe – Whether the content and language style is understandable to our community. Does it give a right message to people with referable level DR?  Probe – What is your opinion of organization of the content, layout and designs? |
| [Delivery] |
| 4) What is your opinion on time taken for delivering the intervention to a service user?  Probe – Does it interrupt the usual patient flow? Does it affect the usual process of consultation of people with diabetes? |
| [Technical difficulties] [Barriers to deliver] |
| 5) What were the difficulties faced by graders / educators in delivering health educational intervention?  Probe – Problems in language, content / terminology, interaction with service users, difficulties in multi-tasking (giving prescriptions, checking blood pressure, DR screening and delivering health educational intervention). |
| [User acceptability as perceived by providers] |
| 6) What were the service users’ responses to the proposed health educational intervention?  Probe - How would you describe the service users’ responses to the intervention?  Do you think it is an innovative method to improve the uptake of assessment and treatment services compared to the conventional method?  [Actionability]  Probe – Do you think that patients will attend to eye clinic following this intervention?  Probe – Are there clear instructions, visual aids, steps to follow? |
| [Usability as an intervention] |
| 7) What is your opinion on acceptability of the intervention in medical clinics in long run considering different characteristics of service users?  Probe – with people with different level of education, different literacy levels, different attitudes towards seeking medical care at the clinic? |
| [Understandability or learnability for the providers] [not for patients] |
| 8) What were the problems associated with learning of health educational intervention (by educators)?  Probe – Are you familiar with medium and mode of delivery?  Do you require training on mode of delivering health educational intervention? |
| [Suggestions] |
| 9) In your opinion will this intervention enhance the performance of provider with regard to educating the service users on DR?  Probe - Would this support comprehensive management of a diabetic patient? |
| 10) What are your suggestions to further improve this intervention? |
